# *Mycobacterium abscessus* resists the innate cellular response by surviving cell lysis of infected phagocytes

**DOI:** 10.1371/journal.ppat.1011257

**Published:** 2023-03-27

**Authors:** Hamadoun Touré, Lee Ann Galindo, Marion Lagune, Simon Glatigny, Robert M. Waterhouse, Isabelle Guénal, Jean-Louis Herrmann, Fabienne Girard-Misguich, Sébastien Szuplewski

**Affiliations:** 1 Université Paris-Saclay, UVSQ, INSERM, Infection et Inflammation, Montigny-Le-Bretonneux, France; 2 Department of Ecology and Evolution, University of Lausanne, and the Swiss Institute of Bioinformatics, Lausanne, Switzerland; 3 Université Paris-Saclay, UVSQ, LGBC, Versailles, France; 4 Assistance Publique-Hôpitaux de Paris, Hôpitaux Universitaires Ile-de-France Ouest, GHU Paris-Saclay, Hôpital Raymond Poincaré, Garches, France; National Institutes of Health, UNITED STATES

## Abstract

*Mycobacterium abscessus* is the most pathogenic species among the predominantly saprophytic fast-growing mycobacteria. This opportunistic human pathogen causes severe infections that are difficult to eradicate. Its ability to survive within the host was described mainly with the rough (R) form of *M*. *abscessus*, which is lethal in several animal models. This R form is not present at the very beginning of the disease but appears during the progression and the exacerbation of the mycobacterial infection, by transition from a smooth (S) form. However, we do not know how the S form of *M*. *abscessus* colonizes and infects the host to then multiply and cause the disease. In this work, we were able to show the hypersensitivity of fruit flies, *Drosophila melanogaster*, to intrathoracic infections by the S and R forms of *M*. *abscessus*. This allowed us to unravel how the S form resists the innate immune response developed by the fly, both the antimicrobial peptides- and cellular-dependent immune responses. We demonstrate that intracellular *M*. *abscessus* was not killed within the infected phagocytic cells, by resisting lysis and caspase-dependent apoptotic cell death of Drosophila infected phagocytes. In mice, in a similar manner, intra-macrophage *M*. *abscessus* was not killed when *M*. *abscessus*-infected macrophages were lysed by autologous natural killer cells. These results demonstrate the propensity of the S form of *M*. *abscessus* to resist the host’s innate responses to colonize and multiply within the host.

## Author summary

*Mycobacterium abscessus*, a fast-growing mycobacterium and an opportunistic pathogen, is a harmful bacterium for people with cystic fibrosis. It can subsist on two morphological forms: rough (R) and smooth (S). R-*M*. *abscessus* results from the irreversible transition of S-*M*. *abscessus*. While severe pulmonary infections are associated with the presence of the R form, S-*M*. *abscessus* is considered the infective form. How S-*M*. *abscessus* resists the host innate response before to establish an infection remains unclear. Using *Drosophila*, we observed that S-*M*. *abscessus* is rapidly internalized by *Drosophila* phagocytic cells and the bacterium grows in these latter. Thanacytes, another *Drosophila* immune cell population, respond to the infection by inducing a caspase-dependent apoptotic death of the infected phagocytes. However, *S-M*. *abscessus* resists this lysis of phagocytes and grows dramatically leading to a bacteremia, causing the death of the infected flies. We confirmed the ability of intracellular S-*M*. *abscessus* to resist the lysis of phagocytes in a mammalian host by infecting primary murine macrophages and bringing them into contact with autologous natural killer cells. In conclusion, our study demonstrates that S-*M*. *abscessus* shares with strict pathogenic mycobacteria, such as *M*. *tuberculosis*, the virulence trait to resist the host innate cytotoxic response.

## Introduction

Mycobacteria are divided into two groups according to their growth rate and their strict pathogenicity to humans and animals [1]. A typical example of slow-growing mycobacteria (SGM) is *Mycobacterium tuberculosis* complex, which is strictly pathogenic to humans and animals, making this their sole reservoir. In contrast, the group of the rapidly-growing mycobacteria (RGM) comprises most of the mycobacterial species, which are predominantly saprophytic and, therefore, non-pathogenic to humans and animals [2]. An exception is *M*. *abscessus*, the most pathogenic of the RGM, responsible for respiratory and mucocutaneous pathologies in humans with or without predisposing factors [3–5]. After several taxonomical proposals, *M*. *abscessus* complex consists of three subspecies: *M*. *abscessus subsp*. *abscessus*, *M*. *abscessus subsp*. *massiliense*, and *M*. *abscessus subsp*. *Bolletii* [6,7]. Indeed, *Mycobacterium chelonae*, the closest species to *M*. *abscessus*, and *Mycobacterium fortuitum*, were formerly classified in the same complex as *M*. *abscessus*.

*M*. *abscessus* possesses several virulence factors (*e*.*g*. phospholipase C) absent from other RGMs such as *Mycobacterium smegmatis* or *M*. *chelonae* [8]. It is able to survive in amoeba [9] and mammalian macrophages by blocking the acidification of their phagosomes [10]. The essential role played by the *M*. *abscessus esx4* secretion system confirms its similarity with *M*. *tuberculosis* in its intracellular behavior [9]. *M*. *abscessus* also has the property of evolving during the course of infection [11] from a smooth (S) infectious form, to an irreversibly rough (R) form by loss of parietal lipids called glycopeptidolipids [8,12,13]. This R form is associated with more aggressive pulmonary disease [11] and with the formation of cords in zebrafish model, whose large size may protect it from ingestion and destruction by professional phagocytes [14]. These properties can be seen as advantages and possible escape mechanisms from surveillance by the host immune system, promoting infection and survival of *M*. *abscessus* in the host.

Zebrafish and mouse models have confirmed, on the one hand, the increased virulence of *M*. *abscessus*, particularly its R form [11,12,14–16]. On the other hand, they allowed to point the critical role played by several cytokines such as IFNγ [17] and TNFα [16–18] for controlling *M*. *abscessus* infection. These models are not susceptible to an infection with the S form, with a progressive elimination or a persistence in mice [19] and zebrafish [14] respectively.

The virulence of the S form has been recently demonstrated in *Drosophila melanogaster* in an intra-abdominal infection model, with death of the *Drosophila* [20,21].

*Drosophila* is a well-established organism model for studying pathophysiology of bacterial infections, such as those with *Listeria monocytogenes* and *Staphylococcus aureus* [22,23]. Its genetic tractability makes it one of the best models to combine functional genetics with immunity. Indeed, to note, Toll-like receptors were discovered in *Drosophila* [24]. In the context of mycobacterial infections, *Drosophila* has mainly been used to model tuberculosis, with *M*. *marinum* infection [25]. As *M*. *tuberculosis* in humans, *M*. *marinum* causes a wasting in *Drosophila* [26], associated with a metabolic switch [27]. *Drosophila* has allowed to highlight the crucial role of the STAT-ATG2 pathway in the control of mycobacterial infections by macrophages [28]. Few studies have been conducted in *Drosophila* with *M*. *abscessus*, mainly to test the efficacy of some drug combinations against *M*. *abscessus* [21].

The availability of a model of susceptibility to infection by the S form has allowed us to study the propensity of *M*. *abscessus* to resist the protective innate responses of the infected host. Indeed, we set up a modified *Drosophila* infection model by administering S *M*. *abscessus* intrathoracically with full control of the injected inoculum. After systemic injection, *M*. *abscessus* was rapidly internalized by phagocytes, allowing it to avoid the antimicrobial peptide response, and with intracellular growth before spreading into the circulation. Our results highlight that M. abscessus resists the lysis and caspase-dependent apoptotic cell death of Drosophila infected phagocytes, which was further confirmed in mice, with murine M. abscessus-infected macrophages lysed by autologous natural killer cells.

## Results

### *M*. *abscessus* is more pathogenic for *Drosophila* compared to some other RGM and SGM

**Fig 1:**
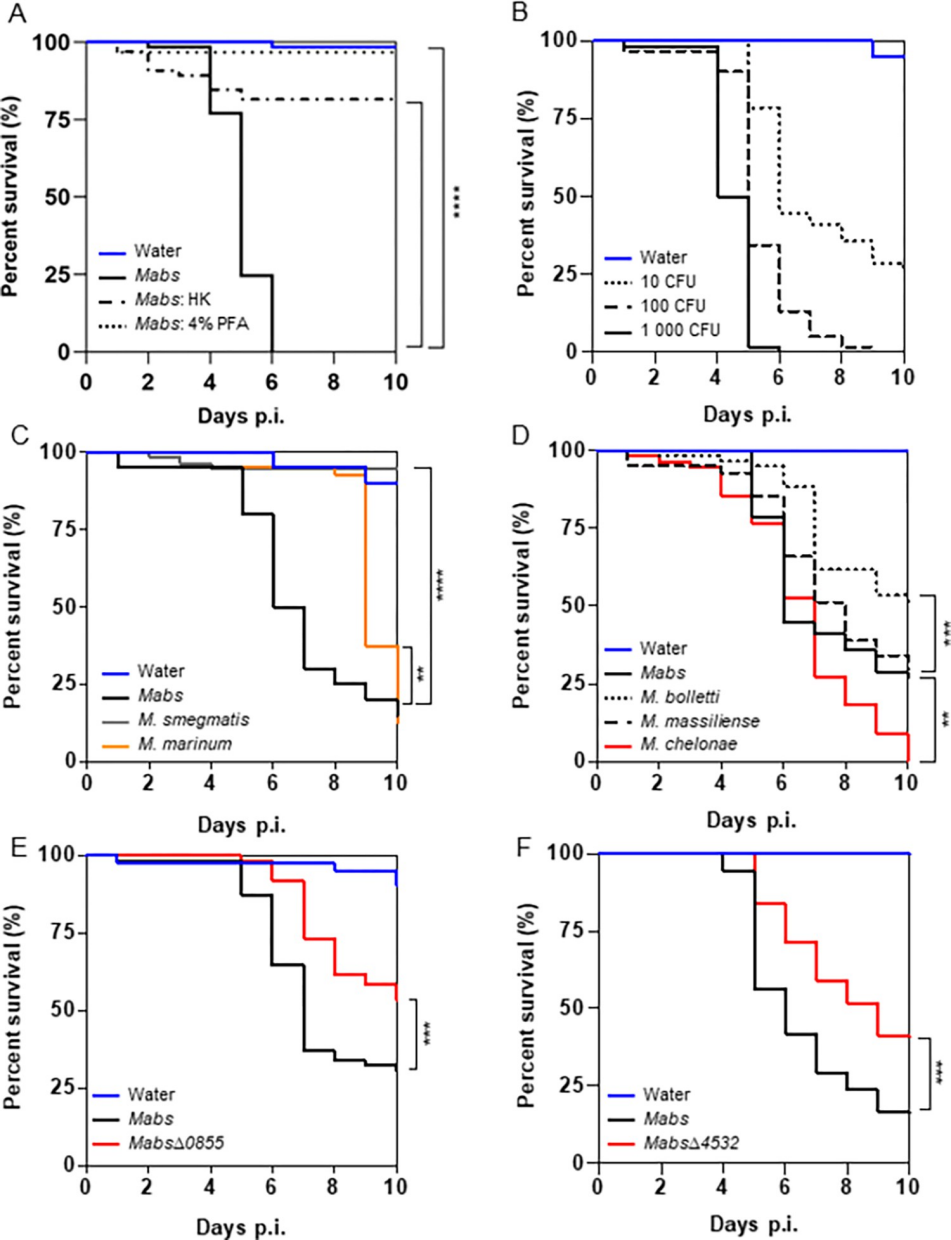
*M*. *abscessus* is virulent in *Drosophila*. (A-F) (A) Survival curves of *w*^*1118*^ flies injected with water or 1,000 CFU (Colony Forming Unit) of living *M*. *abscessus* (*Mabs*), heat-killed *M*. *abscessus* (*Mabs*: HK) or *M*. *abscessus* fixed with 4% PFA (*Mabs*: 4% PFA) (B) different doses of live *M*. *abscessus* (10, 100 or 1,000 CFU) (C) 10 CFU of different mycobacterial species of different virulence (*M*. *abscessus*, *M*. *marinum* or *M*. *smegmatis*) (D) 10 CFU of different subspecies of *M*. *abscessus* and *M*. *chelonae* (E-F) 10 CFU of *M*. *abscessus* and the *M*. *abscessus Δ0855* mutant strain (E) and *Δ4532* mutant strain (F), both defective for intracellular growth. The curves represent the survival of 60 flies. The data were analyzed using the log-rank statistical test. Asterisks represent p values **p <0.01, ***p <0.001, ****p <0.0001.

Thoracic nano-injections were performed with different doses of S *M*. *abscessus*. All flies injected with 1,000 colony-forming-units (CFU) of S *M*. *abscessus* died after 6 days post-infection (p.i.). A nearly complete absence of fly death was observed when S *M*. *abscessus* heat- or PFA-killed were injected (Fig 1A). At the same time point (6 days p.i.), 50% of lethality was observed for the lowest dose (10 CFU) (Fig 1B), compared to 87% of flies infected with 100 CFU, showing that death of infected flies was concentration dependent.

We next infected flies with 1,000 CFU of S and R *M*. *abscessus*. 50% of R *M*. *abscessus* infected-flies died on day 3 p.i. whereas the median survival of S *M*. *abscessus* infected flies was delayed to day 5 p.i. (S1 Fig), suggesting that R *M*. *abscessus* is more virulent than S *M*. *abscessus* in *Drosophila*.

Comparison of fly survival after infection with other mycobacteria at the 10 CFU dose confirmed the acute virulence of S *M*. *abscessus* in *Drosophila* (Fig 1C), with delayed death when flies were infected by *M*. *marinum*, a strict pathogenic SGM for humans, or absence of death when flies were infected by *M*. *smegmatis*, a saprophytic RGM (Fig 1C).

We also compared the survival of flies infected with subspecies of *M*. *abscessus* and with *M*. *chelonae* (Fig 1D). *M*. *chelonae* was the most virulent, killing all infected flies on day 10 p.i. whereas *M*. *abscessus subsp bolletii* was the least “pathogenic”, killing only half of the population at the same time point. *M*. *abscessus subsp massiliense* behaved in the same way as S *M*. *abscessus* (Fig 1D).

Finally, we infected flies with mutant strains of *M*. *abscessus* that we have isolated or generated and validated in our previous works for their attenuation in intracellular growth in cellular and/or zebrafish models [9,29,30]. Thus, the *mmpL8_*_*MAB*_ mutant is characterized by an impaired adhesion to macrophages, a decreased intracellular viability, a delay in making cytosol/phagosome contact and an attenuated virulence in zebrafish [29]. The *MAB_4532c* is also strongly impaired in intracellular viability and is unable to induce phagosomal membrane damage and to prevent reactive oxygen species (ROS) production by macrophages [30]. Injection of each of these mutants caused a lower mortality as compared to wild-type *M*. *abscessus* infection (Fig 1E and 1F). We also infected flies with three *M*. *massiliense 43S* mutants strains, known to have a transposon (Tn) insertion in a gene of the ESX-4 locus and to be impaired in intracellular growth [9]. Two mutants correspond to Tn insertions in *eccC* and *eccE* genes, encoding for two ESX-4 structural proteins, and the third mutant correspond to a Tn insertion in the gene encoding for the ESX-secreted protein espI. All the 3 Tn mutants were less virulent than the control (S1B Fig).

Altogether, these results confirmed the sensitivity of *Drosophila* to S *M*. *abscessus* infection, which is related to bacterial virulence, as demonstrated by the behavior of *M*. *abscessus* mutants.

This has enabled us to investigate how S *M*. *abscessus* resists the host innate response, allowing it to colonize and ultimately trigger an infection which kills the flies.

### *M*. *abscessus* infection induces an inefficient antimicrobial peptide-based response in *Drosophila*

*Drosophila* innate immune response relies on both acellular and cellular responses. The acellular response, named humoral by drosophilists, is mainly based on the production of antimicrobial peptides (AMPs) and mediated by two conserved NFkB pathways, Toll and immune deficiency (Imd). The cellular response relies on immune blood cells, called hemocytes, among which more than 90% are macrophages (plasmatocytes) [31].

**Fig 2:**
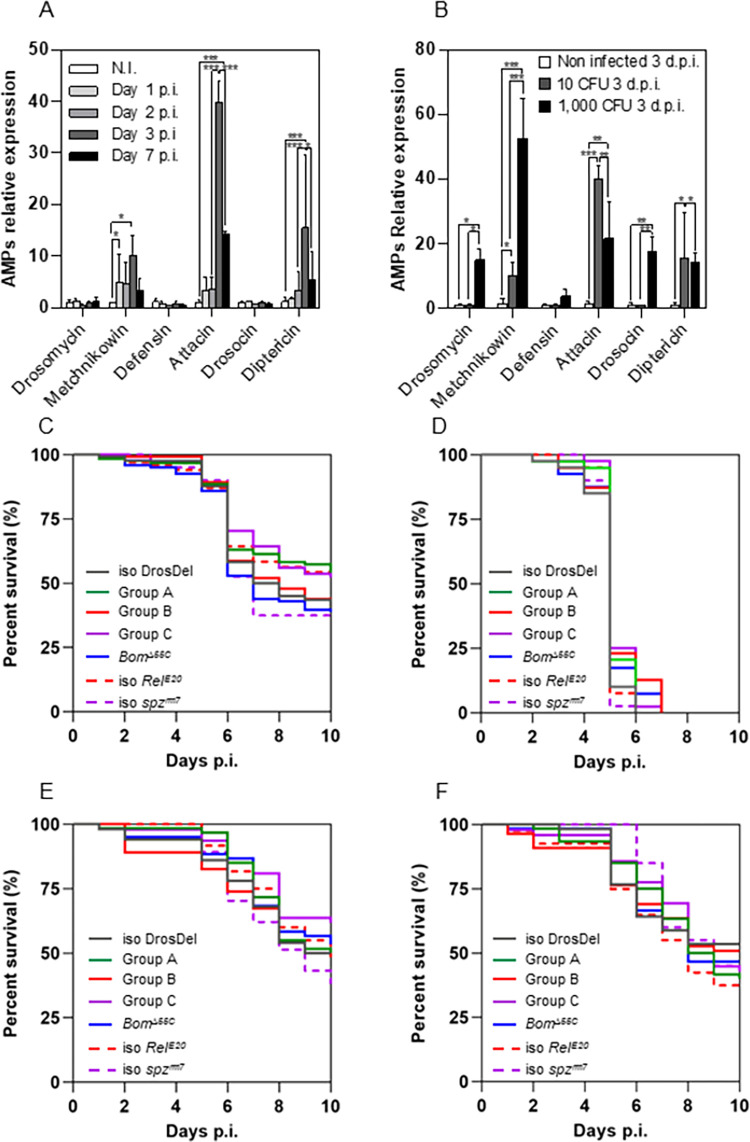
*M*. *abscessus* resists the *Drosophila* AMP response. (A-B) Quantification of relative expression of AMP-encoding genes by qRT-PCR. (A) RNAs were extracted on days 1, 2, 3 and 7 p.i. (post-infection) from *w*^*1118*^ flies injected with 10 CFU (Colony Forming Unit) (B) RNAs were extracted on day 3 p.i. (post-infection) from *w*^*1118*^ flies injected with 10 or 1000 CFU. (C-D) Survival curves of *w*^*1118*^ (iso DrosDel), *Defensin* (Group A), *Attacins*-*Drosocin*-*Diptericin* (Group B), *Drosomycin*-*Metchnikowin* (Group C), *Bomanins* (*Bom*^*Δ55C*^), *Relish* (iso *Rel*^*E20*^) and *spatzle* (*spz*^*rm7*^) mutant flies injected with (C) 10 CFU or (D) 1,000 CFU of *M*. *abscessus*. (E-F) Survival curves of *w*^*1118*^ flies injected with (E) 10 CFU of *M*. *abscessus* and *M*. *abscessus Δ0855* or (F) 10 CFU of *M*. *abscessus* and *M*. *abscessus Δ4532* mutant. Histograms represent data from 3 independent experiments performed in triplicate and error bars represent the standard deviations. Data were analyzed using two-way analysis of variance (ANOVA) (*p*<*0.05; **p*<*0.005; ***p*<*0.0005) in (A-B). Survival was analyzed on 120 flies per genotype in (C) and for 60 flies per genotype in (D-F). Data were analyzed using the log-rank test (*p *=* 0.0475).

First, we studied whether S *M*. *abscessus* infection induced the Imd- and/or Toll-regulated *Drosophila* humoral responses, by quantifying the transcription of the main AMPs-encoding genes, during the course of infection. Injection of 10 CFU of S *M*. *abscessus* resulted in increased levels of the Imd-regulated *Attacin-A* and *Diptericin* transcripts, and of the Toll-regulated *Metchnikowin*, as compared to controls (Fig 2A). This induction peaked on day 3 p.i. Injection of 1,000 CFU of S *M*. *abscessus* resulted in higher transcript levels for almost all Imd- and Toll-regulated genes (Fig 2B). As a control, the wound resulting from nano-injection of water did not induce any expression of AMP-encoding genes on days 0 and 3 (S2A Fig).

We then infected AMP deficient flies generated by CRISPR/Cas9 gene editing technology [32]. These flies were defective in genes encoding AMP regulated by either the Imd pathway (group B:*AttC*^*Mi*^,*Dro-AttA-B*^*SK2*^,*DptA-B*^*Ski*^*;AttD*^*Ski*^), or the Toll pathway (group C: *Mtk*^*R1*^*;Drs*^*R1*^ and Bomanins: *Bom*^*Δ55C*^) or both (group A: *Def*^*SK3*^). These mutant flies were no more sensitive than wild-type controls to water injection (S2B Fig). Mutant flies for *Relish* (*Rel*^*E20*^) and *spatzle* (*spz*^*rm7*^) genes were used as control for the Imd and the Toll pathway respectively. Mutant flies were infected with low and high doses of S *M*. *abscessus* (10 and 1,000 CFU). We did not observe differences in terms of fly survival regardless of the AMP pathway impacted, with an equivalent mortality for all mutated flies (Fig 2C and 2D).

Similar experiments with the same altered flies, but this time performed with the attenuated *M*. *abscessus* mutants, also showed no difference in fly survival, regardless of the AMP pathway impacted (Fig 2E and 2F). Comparatively, the same AMPs mutant flies died when infected with 10 CFU of *B*. *cepacia*, a Gram-negative bacterium (S2C Fig).

Taken together these results show that S *M*. *abscessus* infection induces expression of AMPs-encoding genes. We also show that the absence of AMPs does not modify the mortality of the flies, as opposed to what was observed with *B*. *cepacia*, suggesting that they do not play a major role in the resistance to infection by S *M*. *abscessus*.

### The cellular response of *Drosophila* to S *M*. *abscessus* infection is critical for fly survival

**Fig 3:**
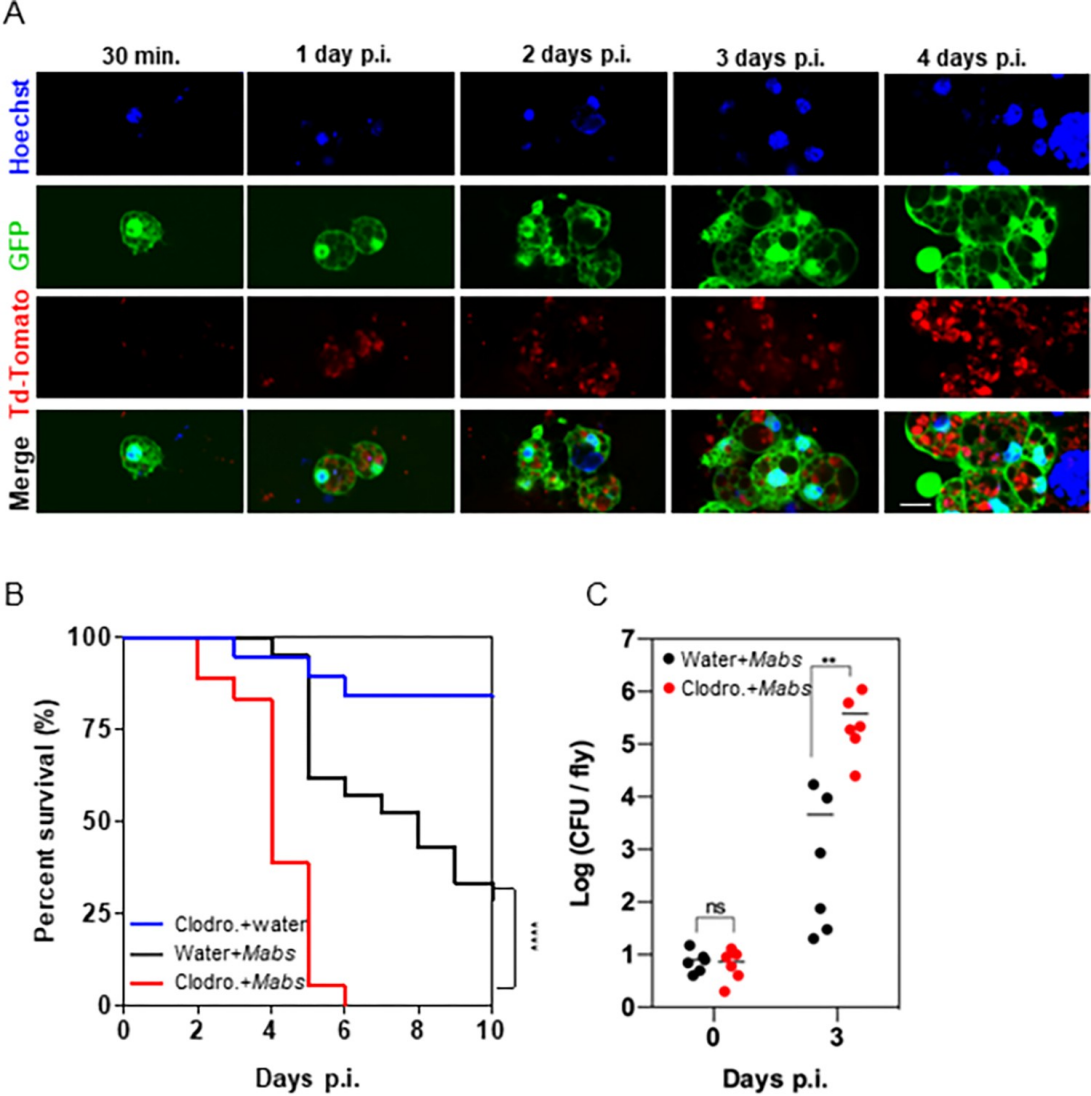
Phagocytes are crucial for controlling *M*. *abscesssus* infection. (A) DNA (Hoechst)-stained hemocytes (GFP) isolated from *hml>eGFP* flies injected with 500 CFU (Colony Forming Unit) of S-*M*. *abscessus* (Td-Tomato) at 30 minutes (min.), and 1, 2, 3, and 4 days post-infection (days p.i). Scale bar represents 5 μm. (B) Survival curves of *w*^*1118*^ flies injected with water or clodronate liposomes (Clodro.) and then injected with water or 10 CFU of *M*. *abscessus* (*Mabs*). (C) Bacterial load quantification on day 3 p.i. by CFU counting of *w*^*1118*^ flies injected with water or clodronate liposomes (Clodro.) and injected with 10 CFU of *M*. *abscessus* (*Mabs*). Survivals were analyzed in 40–60 flies per condition using the log-rank test (*p*<*0.05; ****p*<*0.0001) (B and D). Bacterial loads were individually quantified from 6 flies per condition. Each point represents the CFU number of one fly, and the horizontal traits represent the mean per condition. Data were analyzed using two-way ANOVA statistical tests. Asterisks represent p-values *p = 0.01, **p = 0.005 (C and E).

The cellular response relies on immune blood cells, called hemocytes. Plasmatocytes represent the majority of total hemocyte population in flies, and thus the cellular part of the protective innate response. To assess whether *M*. *abscessus* was internalized by phagocytic plasmatocytes during the course of infection, we used 500 CFU of Td-Tomato fluorescent S *M*. *abscessus* to infect reporter flies with GFP-producing plasmatocytes (*hml>GFP*) [33,34]. Red fluorescent mycobacteria were observed inside GFP-producing plasmatocytes as early as 30 min. p.i. (Fig 3A), and up to 24 hours p.i. Increased red fluorescence, observed up to 4 days p.i. (Fig 3A), indicates that *M*. *abscessus* survives and potentially grows inside the GFP-producing plasmatocytes. Moreover, by looking at the whole fly, up to 5 days p.i., we observe that S *M*. *abscessus* had actively multiplied because it was present in a diffuse way throughout the whole body of the fly (S3A Fig). In comparison, infection with 500 CFU of mCherry fluorescent R *M*. *abscessus* led to intra-plasmatocyte and extracellular growth during the 3 first days of the infection (S3B Fig). This ends up with plasmatocytes’ explosion (S3B and S3C Fig) and formation of mycobacterial cords (S3D Fig) in the hemolymph (blood equivalent in *Drosophila*) on day 3 p.i.. This resulted to a disseminated bacteriemia as early as 3 days p.i. (S3E Fig), leading to the death of all infected flies on day 4 p.i..

The progressive effect of the S *M*. *abscessus* infection, with a potential dissemination only at the 4th - 5th day, might indicate a protective role of plasmatocytes in the control of the infection, at least at its beginning. To test this hypothesis, we treated flies with clodronate containing liposomes. This treatment, depleting phagocytic plasmatocytes [35] (S4 Fig), led to a very high mortality rate, with all clodronate pre-injected flies dying on day 6 p.i. as compared to control flies (Fig 3B), and was associated with a significant increase in S *M*. *abscessus* growth (Fig 3C).

To confirm the critical role of phagocytic plasmatocytes on the infection control, we used the *UAS/GAL4* system to perform genetic depletion of these immune cells, as previously described [36,37]. Indeed, the *UAS/GAL4* system allows the spatial and temporal control of transgene expression. It is based on the use of the yeast transcription factor, Gal4. The binding of this transcriptional activator on a minimal regulatory sequence, called *UAS* (*Upstream Activating Sequence*) drives the expression of a sequence located downstream of this UAS sequence [38]. Here, we used transgenic *UAS-debcl* flies, allowing a Gal4-dependent expression of a proapoptotic gene, in order to kill the cellular populations of interest. To drive its expression in plasmatocytes, we crossed UAS-debcl flies with transgenic Hemese (He)-GAL4 or croquemort (crq)-GAL4 plasmatocytes driver lines. He and crq correspond to transcriptional enhancer controlling expression of plasmatocytes markers and so the expression of the GAL4 gene in these driver lines. Therefore, flies carrying both transgenes (driver and UAS-debcl) express debcl pro-apoptotic gene in their plasmatocytes.

**Fig 4:**
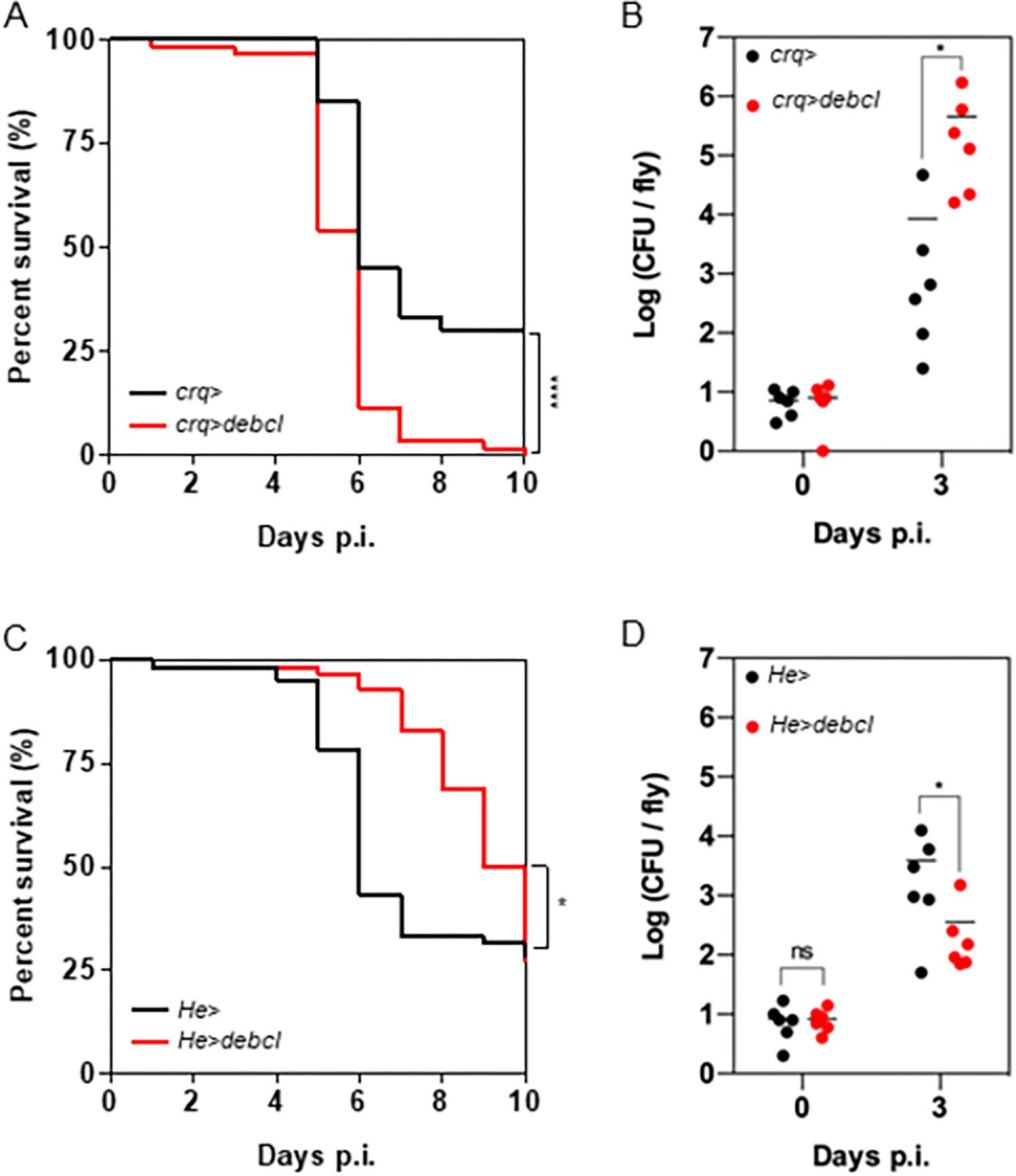
Plasmatocytes sub-populations have opposite contribution during *M*. *abscessus* infection. (A) Survival curves of *crq>* and *crq>debcl* flies injected with 10 CFU (Colony Forming Unit) of *M*. *abscessus*. (B) Bacterial load quantification on 0 and 3 days post-infection (Days p.i.) by CFU counting of *crq>* and *crq>debcl* flies injected with 10 CFU of *M*. *abscessus*. (C) Survival curves of *He>* and *He>debcl* flies injected with 10 CFU of *M*. *abscessus*. (A and C) Survivals were analyzed on 60 flies per genotype using long-rank test (*p *=* 0.03; ****p*<*0.0001). (B and D) Bacterial loads were individually quantified from 6 flies per genotype. Each point represents the CFU number of a fly, horizontal traits representing the mean CFU of a condition. (B and D) Data were analyzed by using a two-way ANOVA test (*p*<*0.05, ns = non-significative).

Flies expressing *debcl* receiving a water injection remained alive throughout the experiments (S5A Fig). Infected control driver flies showed a similar survival rate to the wild-type flies (S5B Fig). Depletion of *crq* expressing cells led to a drastic increase of fly mortality of *M*. *abscessus* infected flies compared to control flies (Fig 4A) and was confirmed by the increased bacterial load on day 3 p.i. for the *crq>debcl* fly genotype (Fig 4B).

Taken together these results demonstrate that phagocytic plasmatocytes rapidly internalized S. *M*. *abscessus*. Their absence makes the flies hyper-susceptible to infection, indicating that these cells play a protective role against S *M*. *abscessus*, at least during the first days of infection.

### S *M*. *abscessus* infection is favored by a deleterious *Drosophila* immune cell population

Surprisingly, depletion of *Hemese (He)* expressing cells conferred an opposite phenotype to *crq>debcl* flies, with increased resistance of the *He>debcl* flies to S *M*. *abscessus* infection and a better control of mycobacterial growth (Fig 4C and 4D).

Several recent works [39–42] tend to indicate that the population of plasmatocytes is more diverse than expected. At least six major populations have recently been defined within larval plasmatocytes [43]. Based on our two opposite phenotypes, we investigated which gene markers were differentially expressed within these different sub-populations. *Hemese*, *Tep4* and *ance* genes have been reported to be more highly expressed than c*rq* gene in a new class of plasmatocytes called thanacytes (as we will refer to them throughout the text for a better understanding) at the larval stage [39]. Interestingly, this population might correspond to the secretory Plasmatocytes, very recently described at the pupal stage, strongly suggesting a persistence of thanacytes in adult flies [44]. We thus confirmed the presence of these cells in adult flies by confocal microscopy by observing Tep4>GFP and ance>GFP positive hemocytes (S6A Fig).

**Fig 5:**
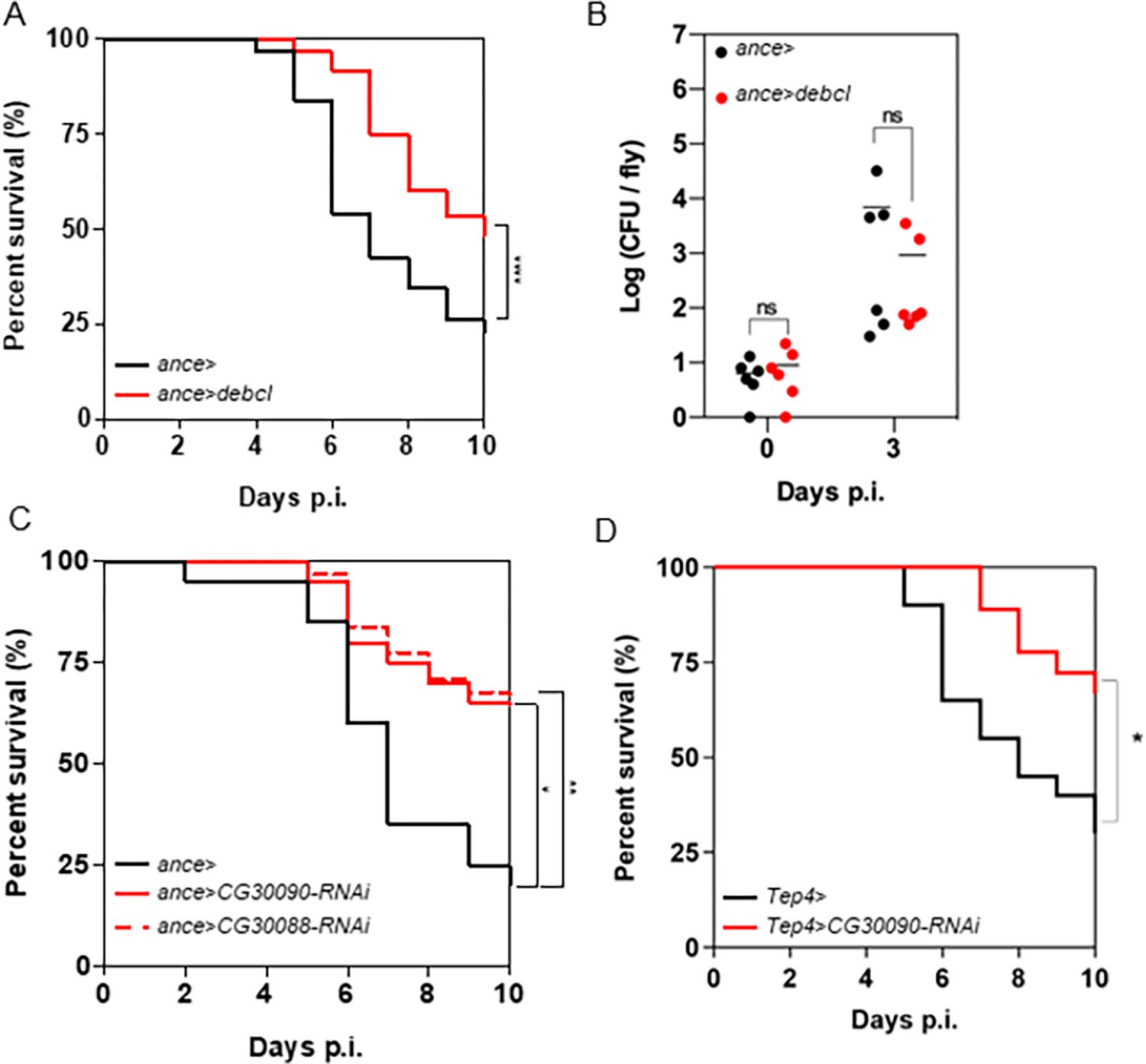
*M*. *abscessus* resists CG30090-mediated lysis of phagocytes by thanacytes. (A) Survival curves of *ance>* and *ance>debcl* flies injected with 10 CFU of *M*. *abscessus*. (B) Bacterial load quantification at 0 and 3 days p.i. by CFU counting of *ance>* and *ance>debcl* flies injected with 10 CFU of *M*. *abscessus*. (C) Survival curves of *ance>*, *ance>CG30090-RNAi* and *ance>CG30088-RNAi* flies injected with 10 CFU (Colony Forming Unit) of *M*. *abscessus*. (D) Survival curves of *Tep4>* and *Tep4>CG30090-RNAi* flies injected with 10 CFU of *M*. *abscessus*. Survivals were analyzed in 40–60 flies per condition using long-rank test (*p*<*0.05; **p*<*0.005; ****p*<*0.0001) (A, C, D). Bacterial loads were individually quantified on 6 flies per condition (B). Each point represents the CFU number of a fly, horizontal traits representing the mean CFU of a condition. Data were analyzed by two-way ANOVA test (ns = non-significant).

By using the same *UAS/GAL4* approach, we looked more specifically at whether depleting thanacytes, by driving *UAS-debcl* by *ance-GAL4*, resulted in a resistance to infection by S *M*. *abscessus*, as observed when we depleted for *He*-positive plasmatocytes. Firstly, *ance>debcl* flies showed an increased survival as compared to controls and CFU counts were not significantly different (Fig 5A and 5B). Similar results were observed when using the TARGET system [45], allowing *debcl* expression only at the adult stage (S6B Fig). In this system, Gal4 activity is inhibited by Gal80 and this inhibition is conditionally restricted to the period starting from the development to the beginning of the adult stage. Finally, no significant difference was observed in AMPs encoding gene expression level between *M*. *abscessus*-infected *ance>* and *M*. *abscessus*-infected *ance>debcl* flies (S6C Fig), suggesting that the increased survival of *ance>debcl* flies was not due to an increased AMPs expression. Moreover, there was no difference in survival between control and *ance>debcl* flies infected with the avirulent RGM *M*. *smegmatis* (S6D Fig).

Taking all of these results together, we confirm the existence of a cell population, potentially the thanacytes, as defined by the drivers used, which, when depleted, protects the flies from S *M*. *abscessus* infection. Since this improved protection cannot be due to AMPs produced during infection, because of the lack of preferential induction of AMPs in the mutated fly compared to the wild type, we wondered what could be their function that makes them deleterious in S *M*. *abscessus* infected flies.

Thanacytes are defined by a high expression of two serine protease encoding genes, *CG30088* and *CG30090*, which have been proposed to be respectively homologous to granzyme B and granzyme H encoding genes, expressed by mammalian Natural Killer (NK) cells and cytotoxic T CD8^+^ lymphocytes [39]. We thus hypothesized that thanacytes, through the expression of *CG30088* and *CG30090*, could lyse infected phagocytic cells without killing the intracellular *M*. *abscessus*, and then, promote *M*. *abscessus* spreading.

We individually depleted *CG30088* and *CG30090* transcripts in thanacytes by RNA interference (*RNAi*), by crossing *UAS-RNAi* lines with thanacytes-GAL4 drivers. We obsreved an increased survival of *ance>CG30088-RNAi* and *ance>CG30090-RNAi* flies as compared to wild-type flies, with 85% and 80% flies still alive on 7-day p.i. respectively (Fig 5C). Similar results were obtained when the transcripts were only depleted in adult thanacytes (S6B Fig). An increased survival was also observed for *Tep4>CG30090-RNAi* compared to control flies (Fig 5D). As a control, we validated the efficiency of these RNAi lines by qRT-PCR. Interestingly, we observed an increase of the quantity of both transcripts upon infection and that both RNAi significantly reduce these increases (S6E and S6F Fig). Collectively, these results, although indirect because they rely on the level of reduction of the cellular serine protease gene expression, still indicate that the thanacytes, through the production of CG30088 and CG30090, are deleterious for *Drosophila* survival during S *M*. *abscessus* infection.

We next assessed whether depletions of thanacytes’ products *CG30088* and *CG30090* could also confer a protection to flies infected with other bacteria. We observed this protective for infection with a strict pathogenic and slow-growing non-tuberculous mycobacterium, *M*. *marinum*, as compared to another fast-growing mycobacterium *M*. *chelonae* and an extracellular Gram-negative bacterium *B*. *cepacia* (S7A–S7C Fig). These results suggest that S *M*. *abscessus* and *M*. *marinum* share a virulence trait linked to a an intrinsic resistance to *Drosophila* serine-protease response, absent in less or non-pathogenic mycobacteria such as *M*. *chelonae* and *M*. *smegmatis*.

### *M*. *abscessus* infection leads to a caspase-dependent apoptosis of fly infected phagocytes

We have produced two essential results leading to the same resistance phenotype when thanacytes are depleted or CG30090/CG30088 transcripts are depleted. Our working hypothesis was that thanacytes, through the expression of *CG30088* and *CG30090*, could lyse infected phagocytic cells without killing the intracellular *M*. *abscessus*, and then, promote *M*. *abscessus* spreading. We then tested whether plasmatocytes infected by S. M. abscessus could be lysed.

**Fig 6:**
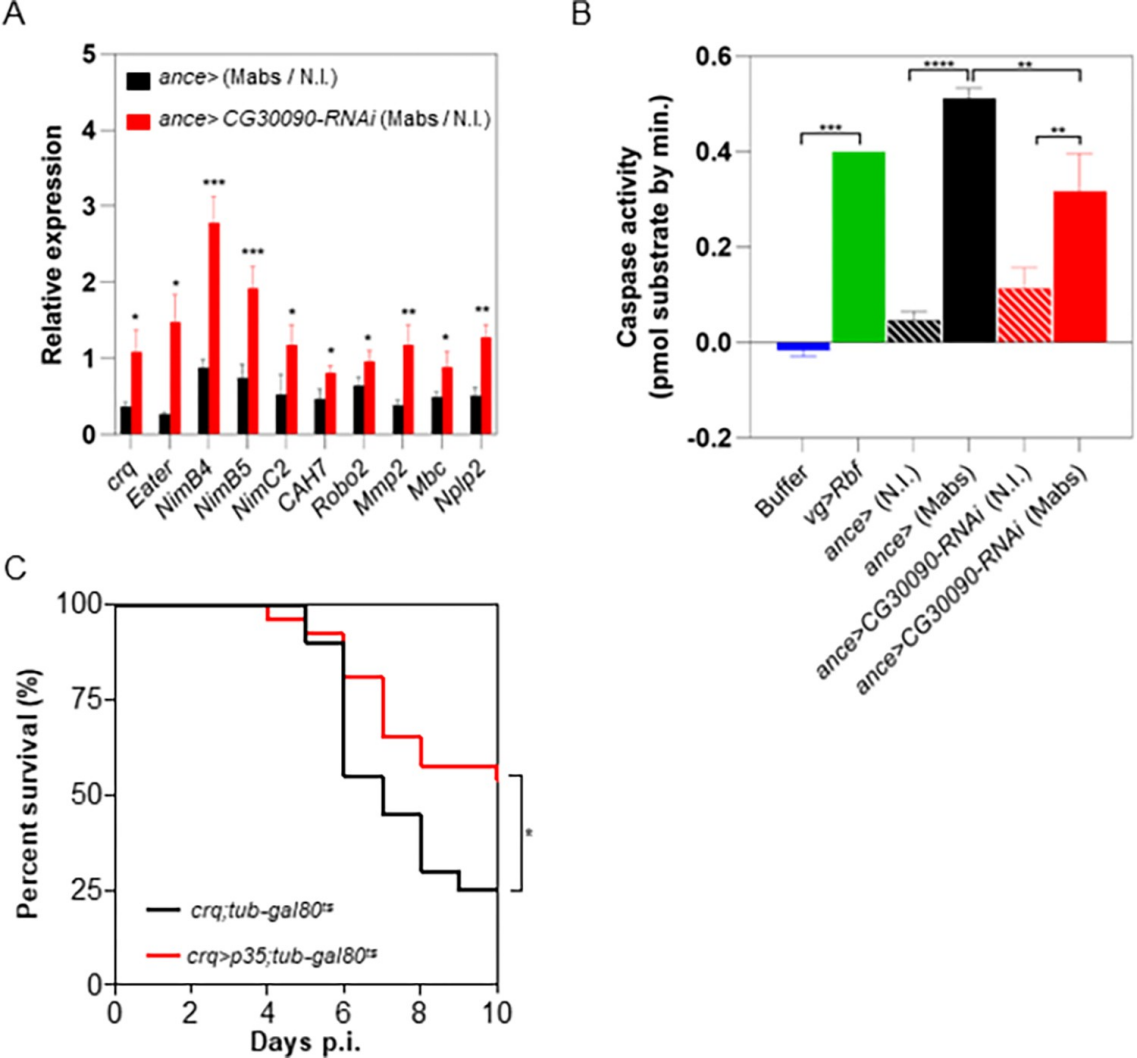
*M*. *abscessus* infection leads to a caspase-dependent apoptosis of phagocytes. (A) Quantification of *crq*, *eater*, *NimB4*, *NimB5*, *NimC2*, *CAH7*, *Robo2*, *Mmp2*, *Mbc* and *Nplp2* genes relative expression by qRT-PCR. RNA were extracted on day 4 p.i. from *ance>* and *ance>GZMH-RNAi* flies injected with water or 10 CFU (Colony Forming Unit). (B) Measurement of caspase activity in protein extracts. Proteins were extracted on day 4 p.i. in hemocytes collected from non-infected (N.I.) or infected (Mabs) *ance>* and *ance>CG30090-RNAi* flies or larval wing disc of *vg>Rbf* flies. (C) Survival curves of *crq>;tub-gal80*^*ts*^ and *crq>p35;tub-gal80*^*ts*^ flies injected with 10 CFU (Colony Forming Unit) of *M*. *abscessus*. Histograms represent data from 3 independent experiments and error bars represent the standard deviations. Data were analyzed using a multiple t-test for (A) and a one-way ANOVA statistical tests (*p*<*0.05; **p*<*0.005; ***p*<*0.0005) for (A-B). Survivals respectively were analyzed on 20 and 27 flies using long-rank test (*p *=* 0.046) in (C).

To do so, we measured the transcript levels of crq and nine additional genes (Mmp2, NimC2, CAH7, Robo2, Mbc, NimB4, NimB5, Nplp2, eater) described as highly expressed in plasmatocytes with phagocytic activity [39,42]. Their abundance would indirectly reflect phagocyte numbers in ance> and ance>CG30090-RNAi flies. On day 4 p.i., relative expression levels of the ten genes were higher in infected ance>CG30090-RNAi flies compared to infected ance> (Fig 6A), relatively to uninfected flies of the two genotypes. These results suggest that the increased expression of these markers during infection, upon serine protease depletion, would be related to a larger phagocytic population, maintained during this infection, unlike the control flies where this expression is reduced. The reduction in the infected phagocytic population might be through caspase-dependent apoptosis [46], with regards to the property conferred to thanacytes via the serine-protease activity [39].

We thus collected infected or uninfected ance> and ance>CG30090-RNAi flies’ hemocytes, extracting their proteins and performing a quantification of caspase activity using a synthetic substrate containing a caspase-3 cleavage site conjugated to a fluorochrome as described in [47]. Protein extracts of larval wing disc of vg>Rbf genotype, known to be apoptotic [48], were used as a positive control. A significant increase of caspase activity was observed in M. abscessus-infected ance> extracts compared to non-infected ones (Fig 6B). Interestingly, this caspase activity significantly decreased when the CG30090 serine protease transcripts were depleted (Figs 6B and S8), showing that the observed caspase activation of infected plasmatocytes is dependent on the products of CG30090.

We then inhibited apoptosis in phagocytes by inhibiting caspase activity. We hypothesized that this should increase Drosophila survival and phenocopy thanacytes and CG30090 transcripts depletions. To test this, we inhibited caspase activation in the adult crq-expressing phagocytic plasmatocytes, by expressing in the latter a transgene encoding the caspase inhibitor baculovirus protein p35. Expression of *p35* in adult phagocytic plasmatocytes significantly increased fly survival compared to control (Fig 6C).

With all of these results taken together, we propose that *M*. *abscessus* infection leads to caspase-dependent apoptosis of the infected phagocytes, leading to the progressive depletion of these latter by thanacytes. This might explain the resistance phenotype of thanacytes or serine-proteases depleted flies, by the maintenance of the phagocytic cell reservoir, the best able to control S *M*. *abscessus* infection. We now have to confirm whether this peculiar trait was also observed in a mammalian host.

### Intracellular *M*. *abscessus* resists lysis of murine macrophages by autologous NK cells

The observed behavior of S *M*. *abscessus* or *M*. *marinum*, as compared to *M*. *smegmatis*, in the fly, led us to evaluate whether, like another slow-growing pathogenic mycobacterium, *M*. *tuberculosis*, we might find the same phenotype of resistance to NK lysis described for *M*. *tuberculosis* [49]. In fact, the CD8^+^ or NK cytotoxic response in human tuberculosis has been shown to be involved in controlling *M*. *tuberculosis* [49]. This response follows two pathways in humans, the granzyme and the perforin-granulysin pathways. *M*. *tuberculosis*, a strict pathogen of humans, is resistant to the granzyme-mediated CD8^+^ and NK cytotoxic response [50]. The observation that the opportunistic S *M*. *abscessus* behaves in a similar way in *Drosophila* obliges us to test this hypothesis of S *M*. *abscessus* resistance to the murine NK response.

**Fig 7:**
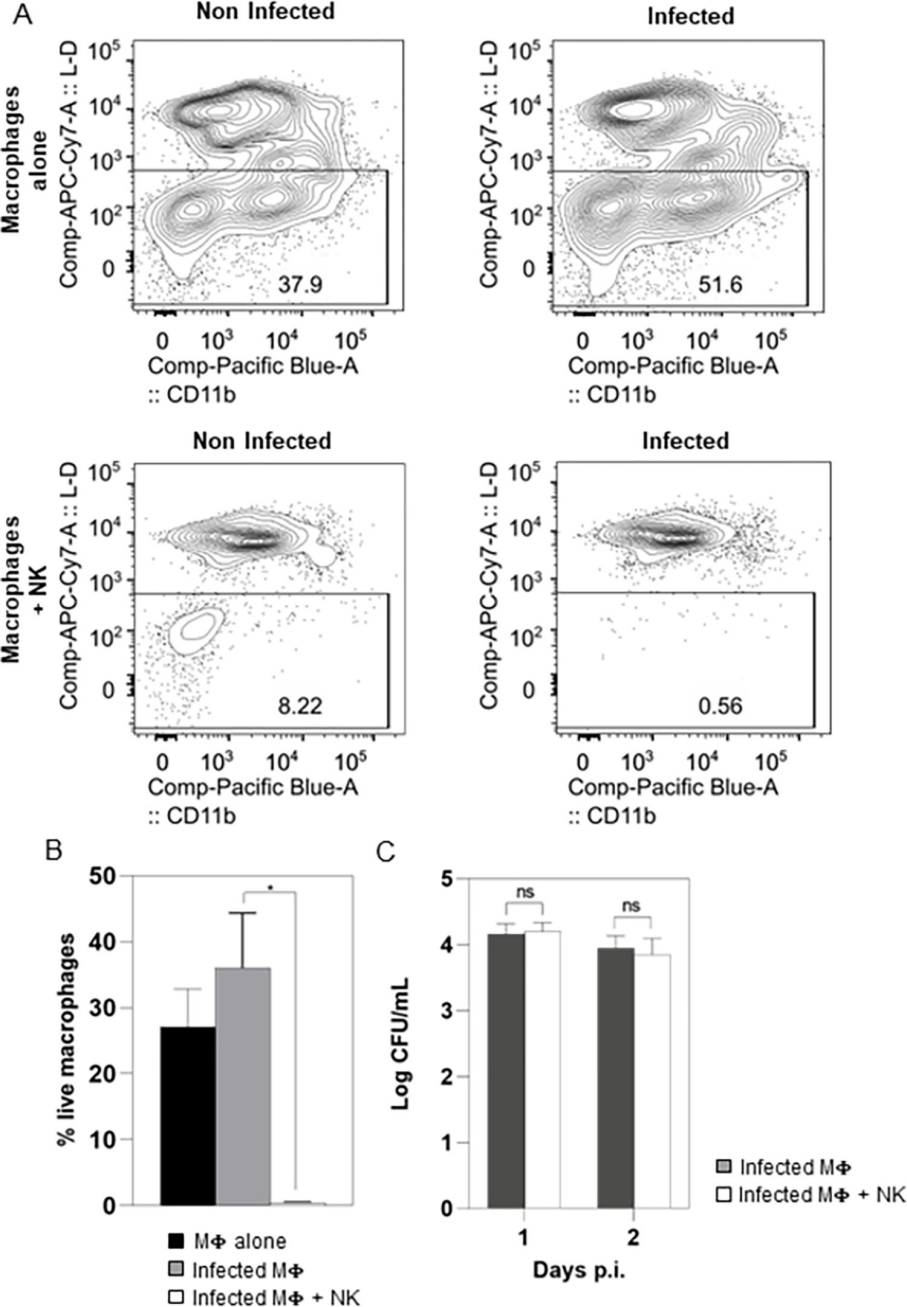
Intracellular *M*. *abscessus* survives the killing of infected macrophages by NK cells. (A-B) Splenic CD11b^+^ macrophages were isolated from naïve mice. Macrophages were either infected or not with *M*. *abscessus*. Then, autologous splenic NK cells were either added or not to the culture. (A) Cells were labelled with live dead staining followed by anti-NK1.1 and CD11b antibody staining. The plots represent the survival of macrophages (NK1.1^-^ cells) that were not infected or 2 days after *M*. *abscessus* infection in the presence or absence of NK cells. (B) Mean survival of macrophages not infected (black bar) or infected with *M*. *abscessus* in the absence (grey bar) or in presence (white bar) of NK cells analyzed by flow cytometry. (C) Intracellular *M*. *abscessus* quantification on day 1 and 2 p.i. by CFU (Colony Forming Unit) counting in the absence (black bar) or in presence (white bars) of NK cells (n = 3 independent experiments) Data were analyzed by Student t test (B; * p<0.05, ns: non-significative) or two-way ANOVA test (C).

To assess whether S *M*. *abscessus* might resist to the lysis of infected macrophages induced by NK cells, we performed co-cultures of purified mouse primary NK cells and autologous macrophages. These latter were infected with *M*. *abscessus* at a MOI of 1:10; then, NK cells were added or not 4 hours p.i. On day 2 p.i., macrophages survival was not decreased by S *M*. *abscessus* infection and even seem to be improved (Fig 7A upper panel and 7B). NK cells addition to non-infected macrophages decreased their survival (Fig 7A lower panel and 7B). This decrease was enhanced when macrophages were infected with S *M*. *abscessus* (Fig 7A lower panel and 7B), reinforcing the critical role of NK cells during infection. Importantly, the bacterial load of intracellular S *M*. *abscessus* was similar both in the presence or absence of NK cells despite the drastic decreased survival of infected macrophages (Fig 7C), corroborating our results in *Drosophila*. Overall, these results show that NK cells can kill S *M*. *abscessus*-infected macrophages, whereas the intracellular mycobacteria are resistant to this lysis.

## Discussion

Over the last decades, in part due to the relative conservation of molecular and genetic pathways of innate immunity with mammals, *D*. *melanogaster* has emerged as a good model among the non-mammalian hosts for studying interactions between host and intracellular pathogens [51,52]. Thus, RNA interference (RNAi) screens, performed on S2 cells (embryonic derived macrophage-like cells), have allowed the dissection of host factors involved in *Legionella pneumophila* and *Listeria monocytogenes* invasion and intracellular replication [53–55] and those required for the entry and survival of *M*. *fortuitum* [56] and *M*. *smegmatis* [57]. Nevertheless, *M*. *marinum*, a strict pathogenic mycobacterium, remains the most studied species *in vivo* in *Drosophila* [58]. In adult flies, this bacterium proliferates within phagocytic plasmatocytes then spreads systemically leading to death [25].

Despite being a RGM, S *M*. *abscessus* survived and proliferated within phagocytic plasmatocytes, similarly to the strict pathogenic SGM *M*. *marinum*. The ability of *M*. *abscessus* to survive in protozoa including amoeba, even after encystment [59], provides it with advantages [60] that might reflect defense mechanisms acquired by the bacterium in contact with these predators, allowing it to survive intracellularly. Moreover, mutant strains unable to proliferate in phagocytic cells were less virulent in *Drosophila*, validating the results obtained in environmental phagocytes (amoebae) and mammalian macrophages [9,30], in which an intracellular growth deficit affects the survival of the *M*. *abscessus* within its host [9,29,30].

*D*. *melanogaster* has the undeniable additional advantage of allowing the analysis of the host’s innate immune response [61]. Unlike infections with *M*. *marinum* or *M*. *smegmatis* in which no AMP production was reported [25], two studies have shown that *Drosophila* infection with S *M*. *abscessus* induces the expression of some AMPs, a Toll related AMP-encoding gene [20,62]. We have confirmed and extended this observation by quantifying the expression of the main AMP-encoding gene transcripts, resulting in the induction of almost all Toll but also Imd-related AMP-encoding genes. Strikingly, when we tested whether the induced AMPs were necessary to control S *M*. *abscessus*, we found that their absence, using *Drosophila* strains lacking AMPs, did not increase fly susceptibility to the infection. Non-mutually exclusive interpretations of these results are either that the cellular response prevails over humoral responses to control S *M*. *abscessus* infection or that the intracellular survival of mycobacteria after internalization by phagocytic cells protects them from the AMPs.

We reveal that *M*. *abscessus* was found in *Drosophila* macrophages (plasmatocytes), where it appears to multiply, although we do not exclude the possibility that plasmatocytes re-internalize extracellular bacteria. *M*. *abscessus* knock-out mutants, impacted in their intracellular survival, are also affected in their ability to grow in *Drosophila*, leading to a parallel between phagocytosis resistance in humans and in *Drosophila*. The importance of phagocytic plasmatocytes is demonstrated by the increased susceptibility of flies observed after either clodronate-mediated depletion of phagocytic cells or genetically mediated depletion of *crq*-expressing plasmatocytes. This population can be considered as the main immune cell type controlling the infection during the first days post-infection but also as an intracellular reservoir for S *M*. *abscessus*.

We have highlighted another sub-population of plasmatocytes, expressing *He*, *ance*, *Tep4*, *CG30088* and *CG30090*, that is detrimental for fly survival. Due to this expression profile, they are presumably the subtype recently identified by single cell sequencing of *Drosophila* larval hemocytes called either thanacytes [39] or PL-Pcd/PL-AMP [42,43], which do not exhibit this capacity for phagocytosis [43] and even more recently described in pupae as Seceretory-PL [44]. Interestingly, *Drosophila* are protected from S *M*. *abscessus* infection when *He-* or *ance*-expressing cells were depleted by expressing the pro-apoptotic *debcl* gene. A similar protection was observed when *CG30088* and *CG30090* transcripts were depleted by RNAi in either *ance-* or *Tep4-*expressing cells. Similar phenotypes were observed with *M*. *marinum* infection. However, no protection was observed in these flies when infected by *M*. *chelonae*, a closely related species to *M*. *abscessus* and *B*. *cepacia*, an extracellular bacterium. This highlights the behavior of *M*. *abscessus*, similar to strict pathogenic mycobacteria towards the host innate cellular response.

Thanacytes, through CG30090 production, induce death of phagocytes through a caspase-dependent apoptosis. However, this cellular response was not effective against the mycobacterium. We confirm this resistance of *M*. *abscessus* to phagocytes cytotoxic lysis in mice. We do not provide a complete mechanism here, but in view of the conserved role of granzymes, our results suggest that the NK-dependent killing of *M*. *abscessus*-infected macrophages may pass through induction of caspase-dependent apoptosis after formation of pores in the cell membrane. This mechanism is consistent with what is observed with *M*. *tuberculosis* infection of dendritic cells. Indeed, NK cells lysed *M*. *tuberculosis*-infected phagocytes. Likewise, they also lysed non-infected activated phagocytes (called bystander cells) depleting the mycobacterial cellular reservoir and thus counterbalancing the inflammatory response at the expense of the host [49,50,63].

Our results support the notion that in both *Drosophila* and murine primary cells, intracellular *M*. *abscessus* resists the host innate cellular response by resisting macrophages’ death. This observation is consistent with pioneering reports on the understanding of T cell cytotoxic responses against *M*. *tuberculosis*. Indeed, human NK cells (defined at the time as double negative lymphocyte for CD4 and CD8) killed *M*. *tuberculosis*-infected macrophages without affecting the viability of the intracellular mycobacteria. This lysis of macrophages by NK cells favors tolerance to the infection, by depleting the mycobacterial cellular reservoir and therefore, reducing the inflammatory response [49]. Likewise, CD8 T cells lysed infected macrophages, but also killed intracellular mycobacteria during this process contrarily to NK cells [49,50]. This *M*. *tuberculosis* destruction during macrophage cytotoxic lysis by CD8 T cells constitutes a mechanism of bacterial control and thus, a protection [49,50,64]. These opposing effects of macrophage lysis by NKs and CD8s on mycobacterial viability promote a balance between the inflammatory response and tolerance to the infection [63,65,66].

To summarize, *Drosophila* infection with S *M*. *abscessus* has allowed us to demonstrate how this mycobacterium escapes the host innate response. Similar to strict human and animal pathogenic SGM, such as *M*. *tuberculosis* or *M*. *marinum*, S *M*. *abscessus* is internalized by phagocytes, within which it survives and seems to replicate. These host cells might constitute a shield from the AMPs response to which *M*. *abscessus* is resistant. Nevertheless, both in *Drosophila* and mice, the resistance of *M*. *abscessus* to the killing of the phagocytes might constitute a mode for mycobacterial spreading and, at least, it might result in a severe depletion of the main cell reservoir able to control *M*. *abscessus* replication. This propensity of *M*. *abscessus* to resist the host innate immune response, typical of strict pathogenic SGM, might partially explain its superior pathogenicity among RGM that are predominantly saprophytic.

## Materials and methods

Key materials used in this study are listed in the Table 1.

**Table 1:** Materials.

REAGENT or RESOURCE	SOURCE	IDENTIFIER
**Antibodies**		
FITC-conjugated anti-mouse NK1.1 (clone NKRP1A)	BD Biosciences	Cat#553164
PE-conjugated anti-mouse CD3 (clone 145-2C11)	BioLegend	Cat#100308
Pacific Blue conjugated anti-mouse CD11b^+^ (M1/70)	BioLegend	Cat#101224
**Bacterial strains**		
*Mycobacterium abscessus subsp abscessus* (*M*. *abscessus herein*)	American Type Culture Collection (ATCC)	*19977*
S *Mycobacterium abscessus* tandem dimer Tomato « tdTomato »	[14]	N/A
R *Mycobacterium abscessus* mCherry	[10]	N/A
*Mycobacterium abscessus Δ0855*	[29]	N/A
*Mycobacterium abscessus Δ4532*	[30]	N/A
*Mycobacterium abscessus subsp bolletii (M*. *bolletii herein)*	*Culture Collection University of Gothenburg (CCUG)*	*50184T*
*Mycobacterium abscessus subsp massiliense (M*. *massiliense herein)*	Collection of Institut Pasteur (CIP)	*108297*
*Mycobacterium abscessus subsp massiliense 43S*	Clinical strain	N/A
*Mycobacterium abscessus subsp massiliense 43S eccC*::*Tn*	[9]	37F10
*Mycobacterium abscessus subsp massiliense 43S eccE*::*Tn*	[9]	14E9
*Mycobacterium abscessus subsp massiliense 43S espI*::*Tn*	[9]	2D10
*Mycobacterium chelonae*	*Culture Collection University of Gothenburg (CCUG)*	47445
*Mycobacterium marinum* wild-type M	Collection of Institut Pasteur (CIP)	64.23
*Mycobacterium smegmatis* (strain mc2 155)	American Type Culture Collection (ATCC)	700084
**Critical commercial assays**		
anti-CD11b microbeads	Miltenyi Biotec	130-049-601
RPMI 1640 media	Gibco, Thermo Fisher Scientific	Cat#11875085
Collagenase D	Roche Diagnostics	11088866001
amikacin	Mylan laboratories Inc.	Cat#B1022
LIVE/DEAD	Thermo Fisher Scientific	Cat#65-0865-14
clodronate liposomes (Clodrosome)	Encapsula	CLD-8901
TRIzol reagent	ThermoFisher	15596026
Turbo DNA-free kit	Invitrogen	AM1907
Superscript III	Invitrogen	18080051
Maxima SYBR green master mix	ThermoFisher	K0221
Pierce BCA protein assay kit	ThermoFisher	
Caspase-3 substrate Ac-DEVD-AFC	VWR	ALX-260-031
**Experimental models: Organisms**		
***Drosophila melanogaster***		
w[*];P{w[+mC] = He-GAL4.*Z}85*	Bloomington Drosophila Stock Center (BDSC)	8699
y[1] w[*];P{w[+mC] = crq-GAL4}2	Bloomington Drosophila Stock Center (BDSC)	25041
y[1] w[*];Mi{Trojan-GAL4.2}Tep4[MI13472-TG4.2]/SM6a	Bloomington Drosophila Stock Center (BDSC)	76750
*w[1118];Mi{GFP[E*.*3xP3] = ET1}Ance[MB09828]*	Bloomington Drosophila Stock Center (BDSC)	27809
*y[1] v[1]; P{y[+t7.7] v[+t1.8] = TRiP.HMJ23926}attP40/CyO*	Bloomington Drosophila Stock Center (BDSC)	62448
y[1] sc[*] v[1] sev[21]; P{y[+t7.7] v[+t1.8] = TRiP.HMC05913}attP40	Bloomington Drosophila Stock Center (BDSC)	65039
w[*];sna[Sco]/CyO;P{w[+mC] = tubP-GAL80[ts]}ncd[GAL80ts-7]	Bloomington Drosophila Stock Center (BDSC)	7018
w[*]; P{w[+mC] = UAS-p35.*H}BH2*	Bloomington Drosophila Stock Center (BDSC)	5073
*hml-GAL4;UAS-EGFP*	Dr. M. Crozatier	[33]
*w* ^*1118*^	Dr F. Rouyer (from NIG fly)	N/A
*UAS-debcl;UAS-debcl*	Dr H. Richardson	[67]
DrosDel *iso w*^*1118*^	Dr B. Lemaitre	[32]
*iso Bom* ^*Δ55C*^	Dr B. Lemaitre	[32]
*iso Rel* ^*E20*^	Dr B. Lemaitre	[32]
Group A (*iso;Def*^*SK3*^)	Dr B. Lemaitre	[32]
Group B (*iso;AttC*^*Mi*^,*Dro-AttA-B*^*SK2*^,*DptA-B*^*Ski*^*;AttD*^*Ski*^)	Dr B. Lemaitre	[32]
Group C (*iso;Mtk*^*R1*^*;Drs*^*R1*^)	Dr B. Lemaitre	[32]
***Mus musculus***		
C57Bl6	Janvier LABS	C57BL/6JRj SPF FEM
**Oligonucleotides for qPCR**		
*Attacin A* forward: CGTTTGGATCTGACCAAGG	Eurogentec	N/A
*Attacin A* reverse: AAAGTTCCGCCAGGTGTGAC	Eurogentec	N/A
*Cecropin C* forward: TCATCCTGGCCATCAGCATT	Eurogentec	N/A
*Cecropin C* reverse: CGCAATTCCCAGTCCTTGAAT	Eurogentec	N/A
*Defensin* forward: GAGGATCATGTCCTGGTGCAT	Eurogentec	N/A
*Defensin* reverse: TCGCTTCTGGCGGCTATG	Eurogentec	N/A
*Diptericin A* forward: GCGGCGATGGTTTTGG	Eurogentec	N/A
*Diptericin A* reverse: CGCTGGTCCACACCTTCTG	Eurogentec	N/A
*Drosocin* forward: TTTGTCCACCACTCCAAGCAC	Eurogentec	N/A
*Drosocin* reverse: ATGGCAGCTTGAGTCAGGTGA	Eurogentec	N/A
*Drosomycin* forward: CTGCCTGTCCGGAAGATACAA	Eurogentec	N/A
*Drosomycin* reverse: TCCCTCCTCCTTGCACACA	Eurogentec	N/A
*Metchnikowin* forward: AACTTAATCTTGGAGCGATTTTTCTG	Eurogentec	N/A
*Metchnikowin* reverse:ACGGCCTCGTATCGAAAATG	Eurogentec	N/A
*RpL32* forward: AGCATACAGGCCCAAGATCG	Eurogentec	N/A
*RpL32* reverse: TGTTGTCGATACCCTTGGGC	Eurogentec	N/A
*CAH7* forward: AGAATCTGCGAATGGTCAACAA	Eurogentec	N/A
*CAH7* reverse: ACCACCACTCAGGGTCAGTT	Eurogentec	N/A
*Robo2* forward: GATCAAACGCCGACACAGAAA	Eurogentec	N/A
*Robo2* reverse: GCTCCGACTTGAGTTAATCCGT	Eurogentec	N/A
*Mbc* forward: ATGTGGGTGATGCGGTCATAA	Eurogentec	N/A
*Mbc* reverse: GCCGCGTATTTCCTTTGCTT	Eurogentec	N/A
*NimC2* forward: CTGGAGACGGGAAAATGCCTG	Eurogentec	N/A
*NimC2* reverse: ACACAGTCGCCATGATAGCAG	Eurogentec	N/A
*Mmp2* forward: AACGACGACCGCATGAAGGTG	Eurogentec	N/A
*Mmp2* reverse: GAAGTGGTTGATCCTTAGCTCCC	Eurogentec	N/A
*Ten-a* forward: CTGCATCCTAGTCCAGGACG	Eurogentec	N/A
*Ten-a* reverse: CATTCTGGCCGTGATCCGA	Eurogentec	N/A
*LpR2* forward: GAAATAGCCTTGCATGTGATTGC	Eurogentec	N/A
*LpR2* reverse: GTGGTAGACGGGATTCTCGAA *NimB4* forward: TTGTGCTCAACTACCGCAAC	Eurogentec Eurogentec	N/A N/A
*NimB4* reverse: CGTCCAGCTCGTATCCCTTA	Eurogentec	N/A
*NimB5* forward: CGTAACGACAACGGTGACTG	Eurogentec	N/A
*NimB5* reverse: GTCTCGTCCAGCTTGTAGCC	Eurogentec	N/A
*Nplp2* forward: ATGGCCAAGCTCGCAATTTG	Eurogentec	N/A
*Nplp2* reverse: CTCAACCTTCTTCGCGTCCA	Eurogentec	N/A
*Eater* forward: GCCCTACTGCAAGGGATGTA	Eurogentec	N/A
*Eater* reverse: GGTGGTTGGATTCAGCTTGT	Eurogentec	N/A
*crq* forward: GAGCCCGATGACGACTTCGACATAT	Eurogentec	N/A
*crq* reverse: ACCCACTTTTTCGTCACAGTCAGCG	Eurogentec	N/A
**Software and algorithms**		
ImageJ	NIH image	N/A
Imaris	Bitplane	N/A
GraphPad Prism 9.0.0	GraphPad Software Inc.	N/A
FlowJo 10.6.2	FlowJo LLC	N/A

### Experimental details

#### Bacterial strains and cultures

All mycobacterial strains were grown at 37°C, except *M*. *marinum* (28°C), and *M*. *chelonae* (30°C), in Middelbrook 7H9 medium (Sigma-Aldrich, Saint-Louis, USA) supplemented with 1% glucose and glycerol 0.2% under aerobic condition until an OD_600_ between 0.6 and 0.8. *B*. *cepacia* was cultured in standard Luria-Bertani (LB) medium. Bacterial cultures were then centrifuged to obtain concentrated aliquots, which were frozen at -80°C in 10% glycerol.

#### *Drosophila* maintenance, crosses and infection

The flies were raised on a standard corn agar medium at 25°C. Crosses were performed at 25°C. An exception is for the TARGET experiments, for which 18°C was used until pupal eclosion and a shift in adults at 29°C. The *UAS-GAL4* system [38] was used to express transgenes. For infections, frozen bacterial aliquots were thawed on ice and homogenized using a 30-gauge insulin needle (Becton-Dickinson, France) to avoid clumps. Serial 10-fold-dilutions were done and 30 μL of each dilution was spread on a blood agar plate for mycobacteria (COS, bioMérieux, France) or on a classic LB agar plate for *B*. *cepacia*. Plates were then stored at 28°C or 37°C for 2 or 3–5 days depending on the bacteria and colony forming unit (CFU) counts were determined.

The bacterial inoculum was diluted with water to obtain a suitable concentration. 5–7 days old virgin female flies were anesthetized with CO_2_ (Inject-Matic, Switzerland), and were infected with 50 nL of the suspension containing 10, 100 or 1,000 bacteria by injection into the sternopleural suture. Infections were performed using a Nanoject III (Drummond Scientific Company, USA) nano-injector charged with a calibrated pulled glass needle made with a DMZ-universal-electrode-puller (Zeitz Instruments, Germany). To saturate phagocytic capacity or chemically deplete the phagocytes, 24h prior to infection with *M*. *abscessus*, flies were individually pre-injected with 50 nL containing 500 polystyrene beads of 1 μm of diameter, or with 69 nL of clodronate liposomes (Clodrosome CLD-8901, Encapsula, USA) diluted in PBS (ratio 1:5), respectively. The flies were anesthetized no more than 10 min. Infected flies were maintained at 28°C under controlled humidity. Twenty flies were used for each experimental condition, and each experiment was performed in at least in three independent replicates. Mortality was recorded daily and surviving flies were transferred into a new vial every two days, until day 10 post-infection (p.i.).

#### Quantification of *M*. *abscessus in vivo* growth

Six infected flies per experimental condition were individually ground in 250 μL of water using sterile polypropylene cones (Kimble 749521–1590, Kimble Chase, USA). The broths were centrifuged at 1,200 *g* for 2 minutes (min.) and diluted by 10-fold serial dilutions. 50 μL of each dilution was spread on VCA3 plates (VCA3, bioMérieux, France) containing selective antibiotics for *M*. *abscessus* (Vancomycin, Colistin, Trimethoprim and Amphotericin B). The plates were maintained at 37°C for one week.

#### Microscopy

To monitor the kinetics of *M*. *abscessus* propagation in the fly body, we infected female wild-type flies with 500 CFU of *M*. *abscessus* Td-Tomato. Living flies were anesthetized daily with CO_2_ and visualized on the dorsal face using a fluorescent stereomicroscope (MZFLIII, Leica Microsystems, Germany) until five days p.i..

To validate plasmatocyte depletion by clodrosome injection, water- and clodrosome-injected *crq> ds-Red* flies were mounted in Washable Clear Glue (Elmer’s) between a slide and a coverslip 24h after injection. Whole flies were imaged using an IX-83 microscope (Olympus) with a 10x objective. Acquisitions were performed using CellSens software and images were reconstituted in 3D using IMARIS software (Bitplane).

Plasmatocyte isolation and observation were performed on 5–7 days old female flies of *hml-Gal4; UAS-eGFP* genotype that were individually infected with 500 CFU of *M*. *abscessus* Td-Tomato. 6–8 flies were dissected as described previously [68]. Circulating and sessile hemocytes were collected in 100 μL of PBS containing 1% Hoechst (Invitrogen Hoechst 33342, USA) at 30 minutes, 24h, 48h, 72h and 96h p.i. 10 μL of the solution were placed between a glass slide and a coverslip and observed under a Leica SP8 X laser scanning confocal microscope. The acquired images were treated with ImageJ software (NIH).

#### qRT-PCR

Total RNA was extracted from 20 female flies per condition using TRIzol reagent (TRI reagent, ThermoFisher, Waltham, USA), chloroform, and isopropanol. Genomic DNA was removed from the extracted RNA using a Turbo DNA-free kit (Invitrogen AM1907, Invitrogen, USA), and cDNA was generated using Superscript III (Invitrogen 18080051, Invitrogen, CA, USA), following the manufacturer’s instructions. qPCR was performed using Maxima SYBR Green Master Mix (ThermoFisher K0221, ThermoFisher, USA), 100 ng of cDNA as a template and 10 μM of target gene-specific primers. Primers used are listed in Table 1. *RpL32* transcript levels were used for normalization and the ΔΔct method was used for relative expression.

#### Caspase activity assay

For each experimental condition, 30–50 female flies were dissected and hemocytes were collected as in [68] in 100 μl of chilled caspase assay lysis buffer (HEPES 50 mM (pH 7.5), NaCl 100 mM, EDTA 1mM, CHAPS 0.1%, sucrose 10%, DTT 5mM, Triton 0.5mM (X-100), glycerol 4%, Protease inhibitor cocktail 1x (cOmplete, Roche)). Hemocytes were collected in 1.5 mL microcentrifuge tubes. Proteins were extracted as previously described [47]. Briefly, hemocytes were homogenized with a handled pestle (5 strokes), lysed by freezing in liquid nitrogen, and rapidly thawed at room temperature 3 times. The lysates were centrifuged at 16,000 x g for 20 min. at 4°C and the supernatant was transferred to a new tube. Protein concentrations were determined using the bicinchoninic acid method (Pierce BCA protein assay kit, ThermoFisher, Waltham, USA) following the manufacturer’s instructions. The caspase activity assay was performed at 37°C in a 96-well plate using 20 μg of protein per condition in 100 μM of the caspase-3 substrate Ac-DEVD-AFC (ALX-260-031, VWR, Radnor, USA) in a total volume of 100 μL, according to the manufacturer’s instructions. Fluorescence was quantified over time using a spectrophotometer (Tecan Infinite M200, Life Sciences) with excitation at 385 nm and emission at 460 nm.

#### Murine immune cells isolation, infection and analysis

Spleens from female C57Bl6 mice were collected and cell suspensions were prepared after a 20 min. treatment with Collagenase D (2 mg/mL, Roche Diagnostics, Switzerland) at 37°C to release macrophages. After red blood cell lysis using Ammonium-Chloride-Potassium lysis buffer, CD11b^+^ cells were isolated by positive magnetic selection, using anti-CD11b microbeads and an AutoMacs Pro Separator according to the manufacturer’s instructions (Miltenyi Biotec, USA). Negative fractions were used to isolate NK cells after staining with FITC-conjugated anti-mouse NK1.1 (clone NKRP1A, BD Biosciences) and PE-conjugated anti-mouse CD3 (clone 145-2C11, BioLegend, USA) (15 min. at 4°C) to exclude NKT cells. Pure NK cells were isolated using a BD Aria III cell sorter (BD-Biosciences, USA).

CD11b^+^ cells were cultured in RPMI 1640 medium (Gibco, Thermo Fisher Scientific, USA) containing 10% heat-inactivated fetal bovine serum (Gibco, Thermo Fisher Scientific, USA). 10^6^ cells in 1 mL per condition were infected with 10^5^ *M*. *abscessus* Td-Tomato (multiplicity of infection of 0.1) (37°C, 5% CO_2_ for 3h). Infected cells were treated for 1h with amikacin (250 μg/mL) to kill extracellular bacteria and then maintained with a lower dose of antibiotic (50 μg/mL) throughout the experiment. Purified NK cells were added to the *M*. *abscessus* infected CD11b^+^ cells at a ratio of 1:2. At days 1 and 2 p.i., cells were labelled, first with LIVE/DEAD staining (Thermo Fisher Scientific, USA), followed by Pacific Blue conjugated anti-mouse CD11b^+^ (M1/70, BioLegend USA) and FITC-conjugated anti-mouse NK1.1 (clone NKRP1A, BD Biosciences, USA). Acquisitions were performed on LSR III Fortessa flow cytometer (BD Biosciences, USA) and data were analyzed using FlowJo software version 10.6.2.

#### Biostatistical analysis

All data were analyzed using GraphPad Prism 9.0.0 (GraphPad Software Inc., USA). The log-rank (Mantel-Cox) test for Kaplan-Meier survival curves was used to evaluate the significance of survival statistics. Quantification of CFU and AMP transcript levels was compared by two-way ANOVA and caspase activity by one-way ANOVA. Comparisons of phagocytic plasmatocyte transcript levels were performed using a multiple Student’s t-test. Statistical significance was set to 0.05.

## Supporting information

S1 Fig *Drosophila* susceptibility is dependent to *M*. *abscessus* virulence. (A-B) (A) Survival curves of *w*^*1118*^ flies injected with 1,000 CFU (Colony Forming Unit) of living smooth *M*. *abscessus* (S-*Mabs*) or rough *M*. *abscessus* (R-*Mabs*). (B) Survival curves of *w*^*1118*^ flies injected with 10 CFU of *M*. *massiliense* 43S or mutated *M*. *massiliense* 43S with transposon in *eccC* (37F10), *eccE* (14E9) or *espI* (2D10) genes. Survivals were analyzed on 60 flies per condition using a long-rank test (**p<0.01, ***p<0.001, ****p*<*0.0001).
(TIF)

S2 Fig AMPs mutant flies are sensitive to *B*. *cepacia* infection. (A) Quantification of AMP-encoding genes relative expression by qRT-PCR. RNAs were extracted on days 0 and 3 from wounded *w*^*1118*^ flies by nano-injection of water. (B) Survival curves of *w*^*1118*^ (iso DrosDel), *Defensin* (Group A), *Attacins*-*Drosocin*-*Diptericin* (Group B), *Drosomycin*-*Metchnikowin* (Group C), *Bomanins* (*Bom*^*Δ55C*^), *Relish* (iso *Rel*^*E20*^) and *spatzle* (*spz*^*rm7*^) mutant flies injected with water or (C) 10 CFU (Colony Forming Unit) of *B*. *cepacia*. Survivals were analyzed on 20 flies per genotype in (A) and 60 flies per genotype in (B) using a long-rank test (****p*<*0.0001).
(TIF)

S3 Fig Visualization of *M*. *abscessus* infection in *Drosophila*. (A-E) (A) Dorsal views of the same live anesthetized *w*^*1118*^ fly injected with water or another one injected with 500 CFU (Colony Forming Unit) of *M*. *abscessus* (*Mabs*), observed with a fluorescent stereomicroscope on day 0, 1, 3 and 5 post-infection (day p.i.). (B) DNA (Hoechst)-stained hemocytes (GFP) isolated from *hml>eGFP* flies injected with 500 CFU (Colony Forming Unit) of R-*M*. *abscessus* (mCherry) at 30 minutes (min.), and 1, 2 and 3 days post-infection (days p.i). Scale bar represents 5 μm. (C) DNA (Hoechst)-stained hemocytes (GFP) isolated from *hml>eGFP* flies injected with 500 CFU (Colony Forming Unit) of R-*M*. *abscessus* (mCherry) on day 3 p.i. Scale bar represents 5 μm. (D) Cord of R-*M*. *abscessus* isolated from *hml>eGFP* flies injected with 500 CFU (Colony Forming Unit) on day 3 p.i. Scale bar represents 10 μm. (E) Lateral views of the same live anesthetized *w*^*1118*^ fly injected with water or another one injected with 500 CFU (Colony Forming Unit) of R-*M*. *abscessus*, observed with a fluorescent stereomicroscope on day 0 and 3 post-infection (day p.i.).
(TIF)

S4 Fig Clodronate injection kills adult Drosophila phagocytes. Lateral view of *crq>ds-Red* flies 24h after injection with water (left) or clodrosome (right) observed with an Olympus IX83 microscope. White spots correspond to red-fluorescent cells.
(TIF)

S5 Fig Hemocytes-depleted flies are viable and do not present an impaired survival after water injection. (A) Survival curves of *w*^*1118*^, *crq>debcl* and *He>debcl* injected with water. (B) Survival curves of *w*^*1118*^, *crq>*, *He>*, *Tep4>* and *ance>* injected with 10 CFU (Colony Forming Unit) of *M*. *abscessus*.
(TIF)

S6 Fig Thanacytes exist in adult flies and have a serine proteases activities. (A) *Tep4-* and *ance*-GAL4 drive an expression in adult *Drosophila* hemocytes. DNA (Hoechst) stained hemocytes (GFP) isolated from *ance>*, *Tep4>*, *ance>eGFP* and *Tep4>eGFP* adult flies. Scale bar represents 5 μm. (B) Survival curves of *ance>;tubgal80*^*ts*^, *ance>debcl;tubgal80*^*ts*^ and *ance>CG30090-RNAi;tubgal80*^*ts*^ flies injected with 10 CFU (Colony Forming Unit) of *M*. *abscessus*. (C) Quantification of AMPs-encoding genes relative expression by qRT-PCR. RNA were extracted on day 3 p.i. (post-infection) from *ance>* and *ance>debcl* flies injected with water or 10 CFU (Colony Forming Unit) of *M*. *abscessus*. (D) Survival curves of *w*^*1118*^ flies injected with water and *w*^*1118*^, *ance>* and *ance>debcl* and *He>debcl* flies injected with 10 CFU (Colony Forming Unit) of *M*. *smegmatis*. (E-F) Quantification of *CG30090* (E) and *30088* (F) genes relative expression by qRT-PCR. RNA were extracted on day 3 p.i. (post-infection) from *ance>*, *ance>CG-30090-RNAi* (E) and *ance>CG30088-RNAi* flies injected with water or 10 CFU (Colony Forming Unit) of *M*. *abscessus*. Survivals were analyzed with a log-rank test and gene expression with one-way ANOVA (*p<0.05, ***p<0.001).
(TIF)

S7 Fig *M*. *abscessus* behaves similar to *M*. *marinum* with regard to cellular responses. (A) Survival curves of *ance>* and *ance>CG30090-RNAi* injected with 10 CFU of *M*. *chelonae* or (B) *B*. *cepacia* or (C) *M*. *marinum*. Survivals were analyzed on 60–80 flies per genotype using a long-rank test (****p*<*0.0001)
(TIF)

S8 Fig Thanacytes induce caspase activation in phagocytes infected with *M*.*abscessus*. Kinetics of A385/A460 relative fluorescence released by the fluorochrome-conjugated caspase-3 substrate Ac-DEVD-AFC after incubation with buffer, *vg>Rbf* larval disc, hemocytes from non-infected (N.I.) or infected (Mabs) *ance>* and *ance>CG30090-RNAi* fly protein extracts on day 4 p.i.. Curves are representative of 3 independent experiments and the error bars represent the standard deviations.
(TIF)
