# Supplementary figures and images for: Mycobacterium abscessus resists the innate cellular response by surviving cell lysis of infected phagocytes
Source: PLoS Pathog. 2023 Mar 27;19(3):e1011257. doi: 10.1371/journal.ppat.1011257 (PMC10079227; doi:10.1371/journal.ppat.1011257)

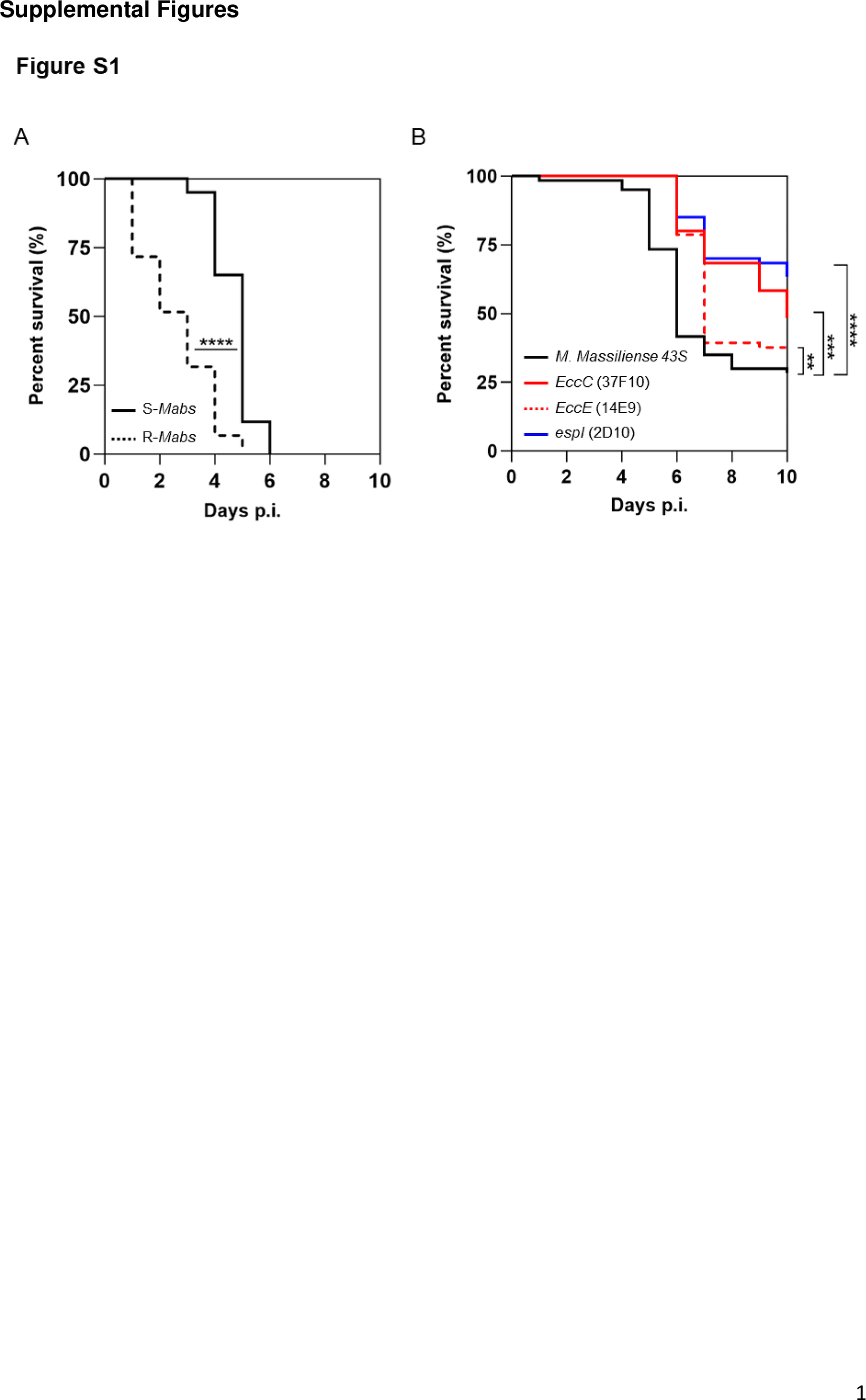

Supplement: S1 Fig — (A-B) (A) Survival curves of w1118 flies injected with 1,000 CFU (Colony Forming Unit) of living smooth M. abscessus (S-Mabs) or rough M. abscessus (R-Mabs). (B) Survival curves of w1118 flies injected with 10 CFU of M. massiliense 43S or mutated M. massiliense 43S with transposon in eccC (37F10), eccE (14E9) or espI (2D10) genes. Survivals were analyzed on 60 flies per condition using a long-rank test (**p<0.01, ***p<0.001, ****p<0.0001). (TIF) [file ppat.1011257.s001.tif]

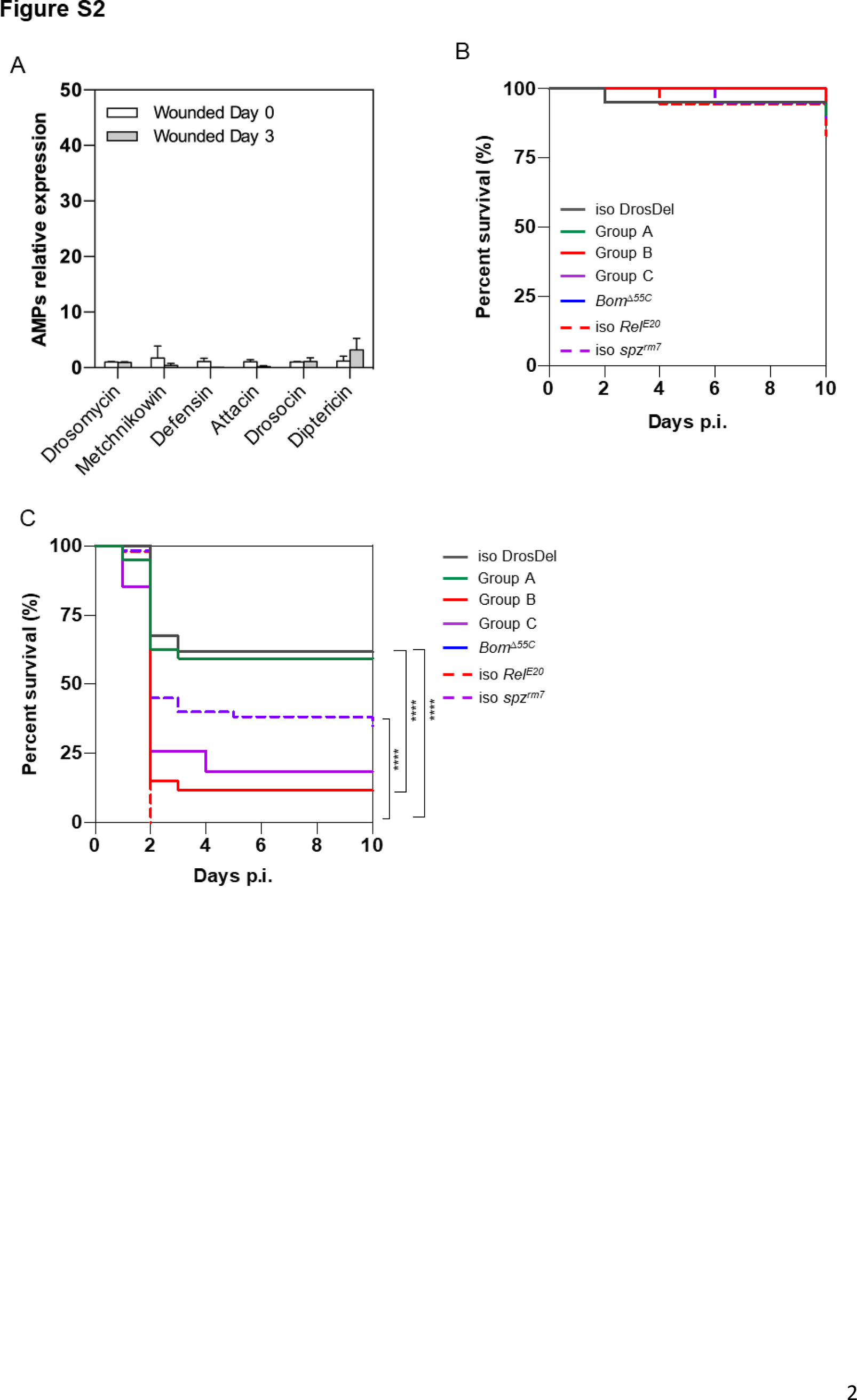

Supplement: S2 Fig — (A) Quantification of AMP-encoding genes relative expression by qRT-PCR. RNAs were extracted on days 0 and 3 from wounded w1118 flies by nano-injection of water. (B) Survival curves of w1118 (iso DrosDel), Defensin (Group A), Attacins-Drosocin-Diptericin (Group B), Drosomycin-Metchnikowin (Group C), Bomanins (BomΔ55C), Relish (iso RelE20) and spatzle (spzrm7) mutant flies injected with water or (C) 10 CFU (Colony Forming Unit) of B. cepacia. Survivals were analyzed on 20 flies per genotype in (A) and 60 flies per genotype in (B) using a long-rank test (****p<0.0001). (TIF) [file ppat.1011257.s002.tif]

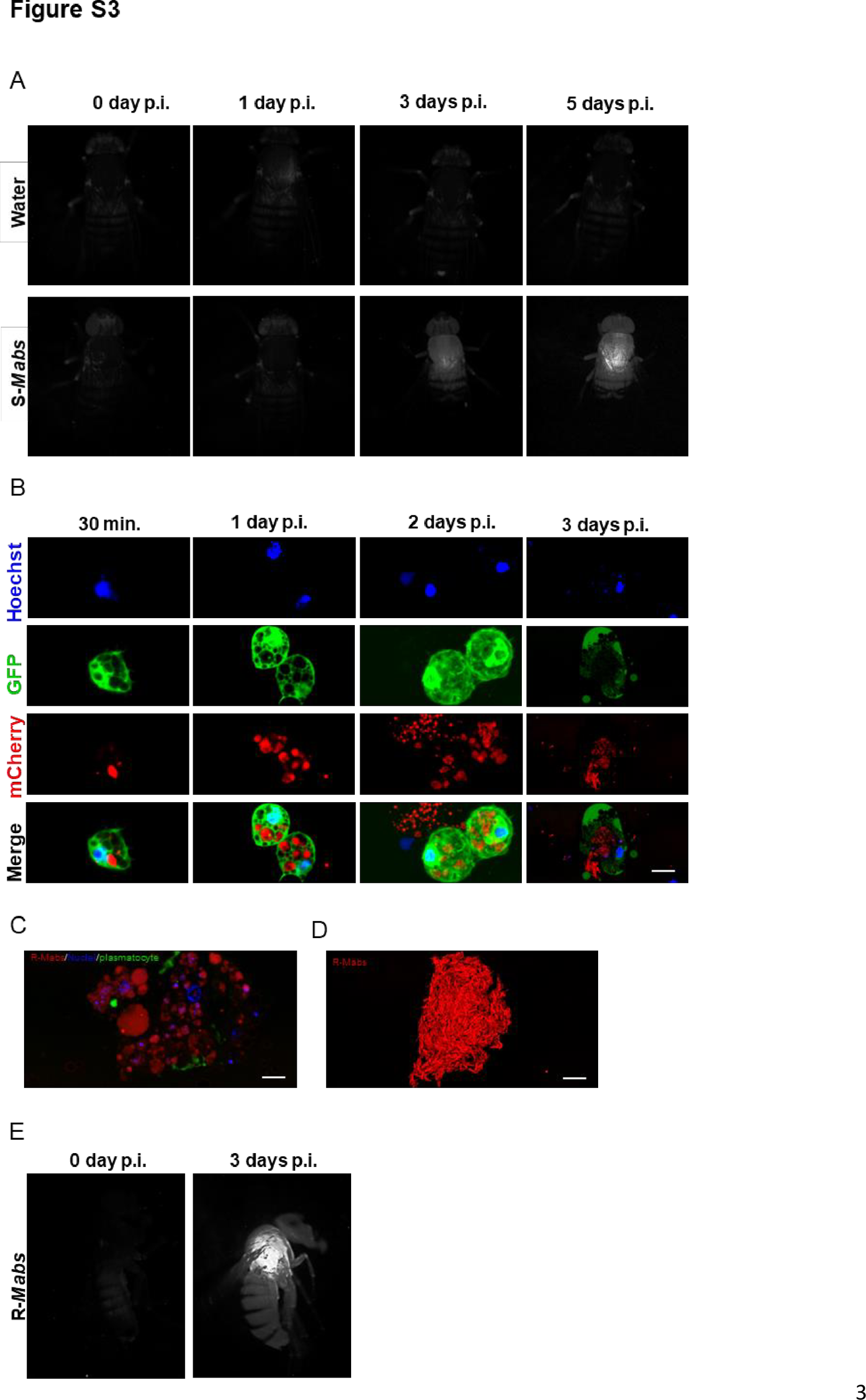

Supplement: S3 Fig — (A-E) (A) Dorsal views of the same live anesthetized w1118 fly injected with water or another one injected with 500 CFU (Colony Forming Unit) of M. abscessus (Mabs), observed with a fluorescent stereomicroscope on day 0, 1, 3 and 5 post-infection (day p.i.). (B) DNA (Hoechst)-stained hemocytes (GFP) isolated from hml>eGFP flies injected with 500 CFU (Colony Forming Unit) of R-M. abscessus (mCherry) at 30 minutes (min.), and 1, 2 and 3 days post-infection (days p.i). Scale bar represents 5 μm. (C) DNA (Hoechst)-stained hemocytes (GFP) isolated from hml>eGFP flies injected with 500 CFU (Colony Forming Unit) of R-M. abscessus (mCherry) on day 3 p.i. Scale bar represents 5 μm. (D) Cord of R-M. abscessus isolated from hml>eGFP flies injected with 500 CFU (Colony Forming Unit) on day 3 p.i. Scale bar represents 10 μm. (E) Lateral views of the same live anesthetized w1118 fly injected with water or another one injected with 500 CFU (Colony Forming Unit) of R-M. abscessus, observed with a fluorescent stereomicroscope on day 0 and 3 post-infection (day p.i.). (TIF) [file ppat.1011257.s003.tif]

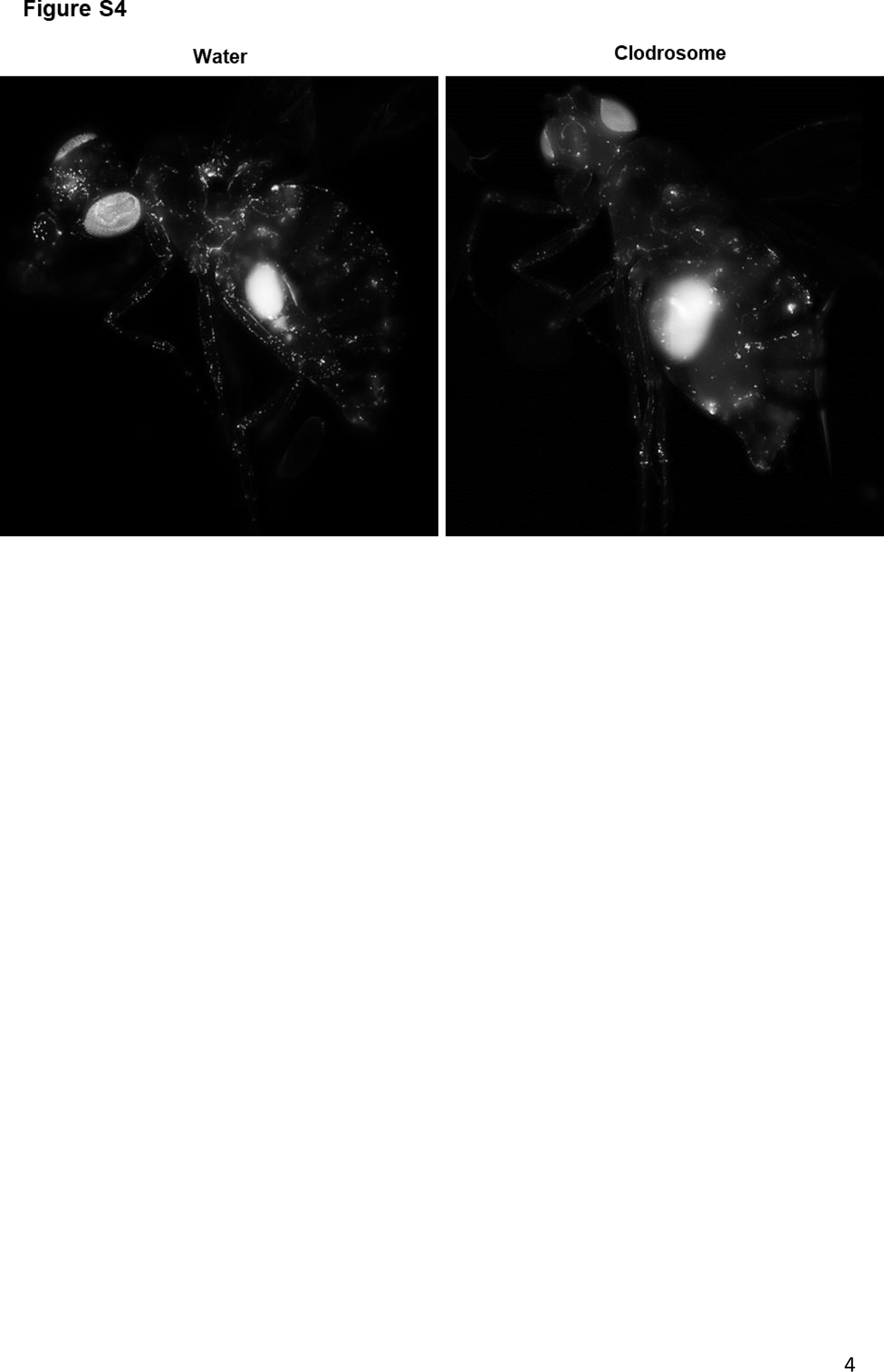

Supplement: S4 Fig — Lateral view of crq>ds-Red flies 24h after injection with water (left) or clodrosome (right) observed with an Olympus IX83 microscope. White spots correspond to red-fluorescent cells. (TIF) [file ppat.1011257.s004.tif]

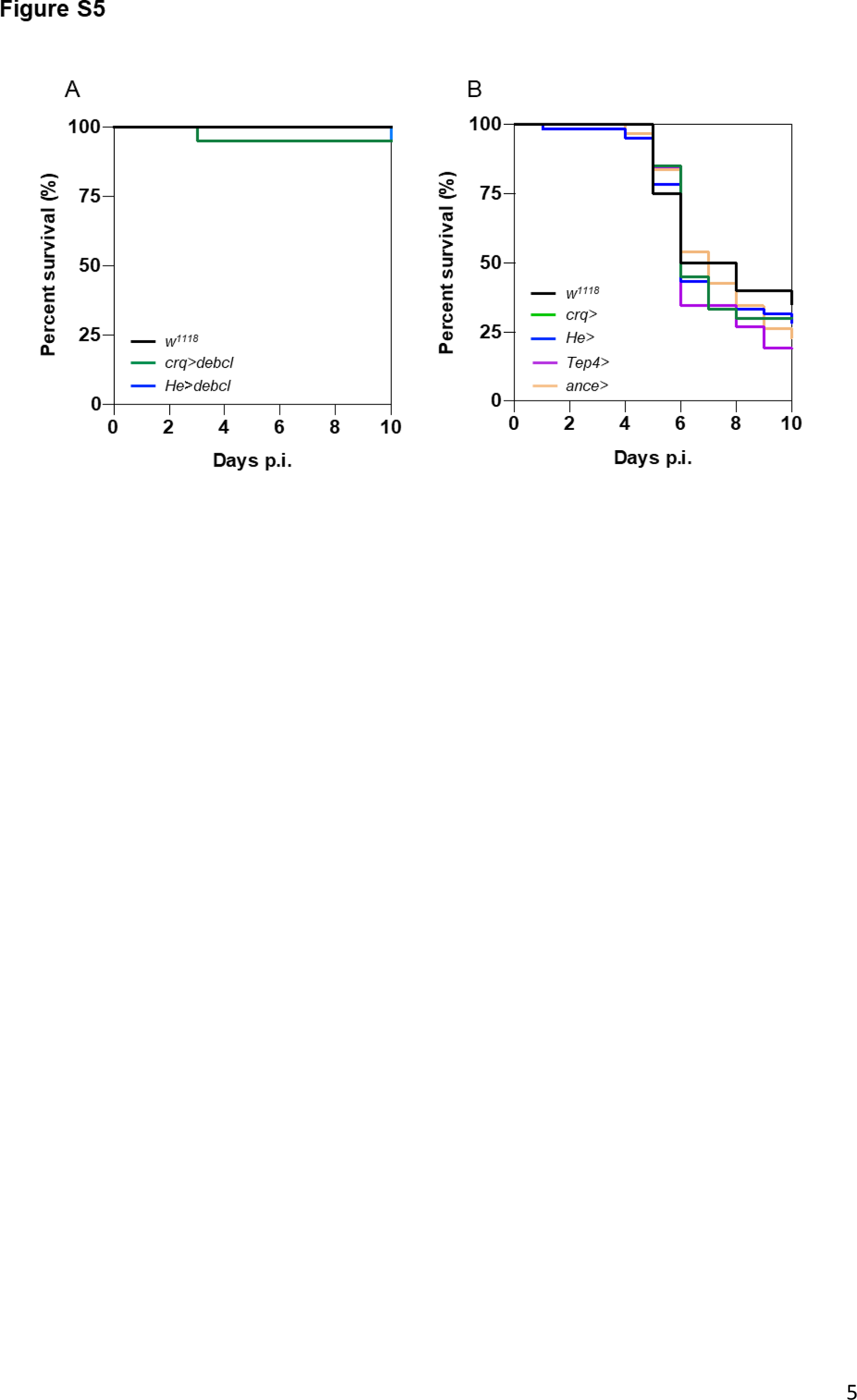

Supplement: S5 Fig — (A) Survival curves of w1118, crq>debcl and He>debcl injected with water. (B) Survival curves of w1118, crq>, He>, Tep4> and ance> injected with 10 CFU (Colony Forming Unit) of M. abscessus. (TIF) [file ppat.1011257.s005.tif]

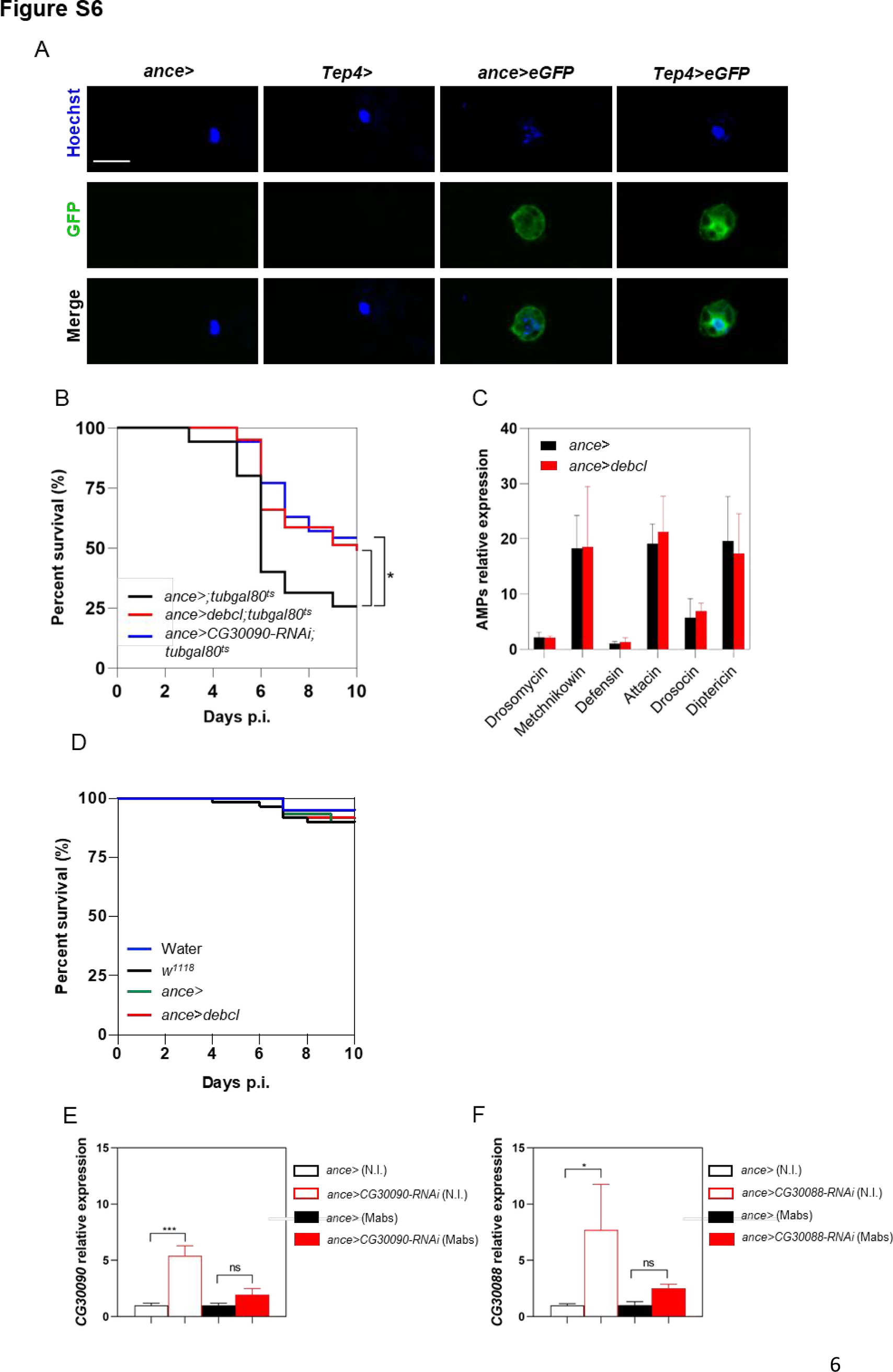

Supplement: S6 Fig — (A) Tep4- and ance-GAL4 drive an expression in adult Drosophila hemocytes. DNA (Hoechst) stained hemocytes (GFP) isolated from ance>, Tep4>, ance>eGFP and Tep4>eGFP adult flies. Scale bar represents 5 μm. (B) Survival curves of ance>;tubgal80ts, ance>debcl;tubgal80ts and ance>CG30090-RNAi;tubgal80ts flies injected with 10 CFU (Colony Forming Unit) of M. abscessus. (C) Quantification of AMPs-encoding genes relative expression by qRT-PCR. RNA were extracted on day 3 p.i. (post-infection) from ance> and ance>debcl flies injected with water or 10 CFU (Colony Forming Unit) of M. abscessus. (D) Survival curves of w1118 flies injected with water and w1118, ance> and ance>debcl and He>debcl flies injected with 10 CFU (Colony Forming Unit) of M. smegmatis. (E-F) Quantification of CG30090 (E) and 30088 (F) genes relative expression by qRT-PCR. RNA were extracted on day 3 p.i. (post-infection) from ance>, ance>CG-30090-RNAi (E) and ance>CG30088-RNAi flies injected with water or 10 CFU (Colony Forming Unit) of M. abscessus. Survivals were analyzed with a log-rank test and gene expression with one-way ANOVA (*p<0.05, ***p<0.001). (TIF) [file ppat.1011257.s006.tif]

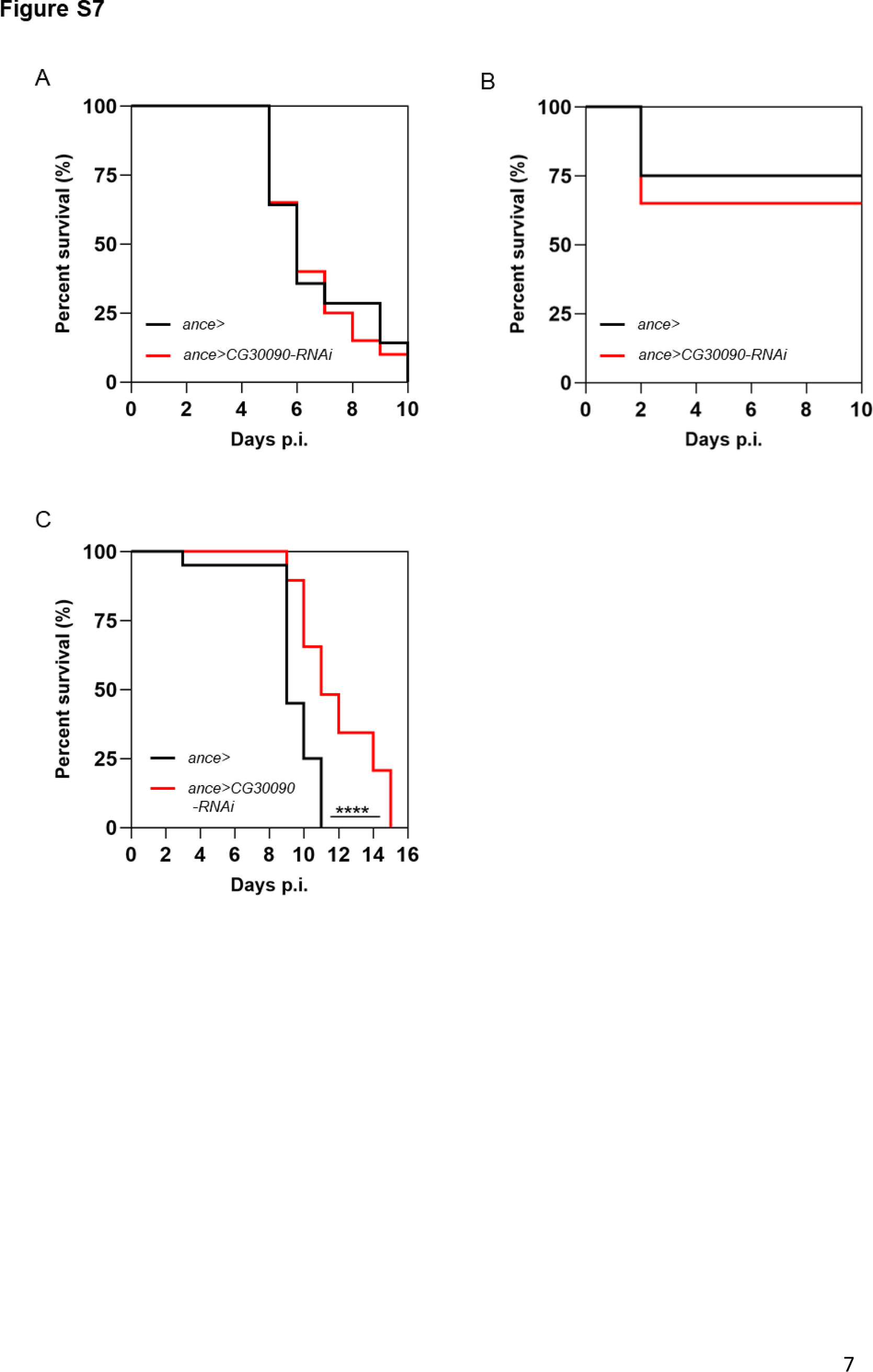

Supplement: S7 Fig — (A) Survival curves of ance> and ance>CG30090-RNAi injected with 10 CFU of M. chelonae or (B) B. cepacia or (C) M. marinum. Survivals were analyzed on 60–80 flies per genotype using a long-rank test (****p<0.0001) (TIF) [file ppat.1011257.s007.tif]

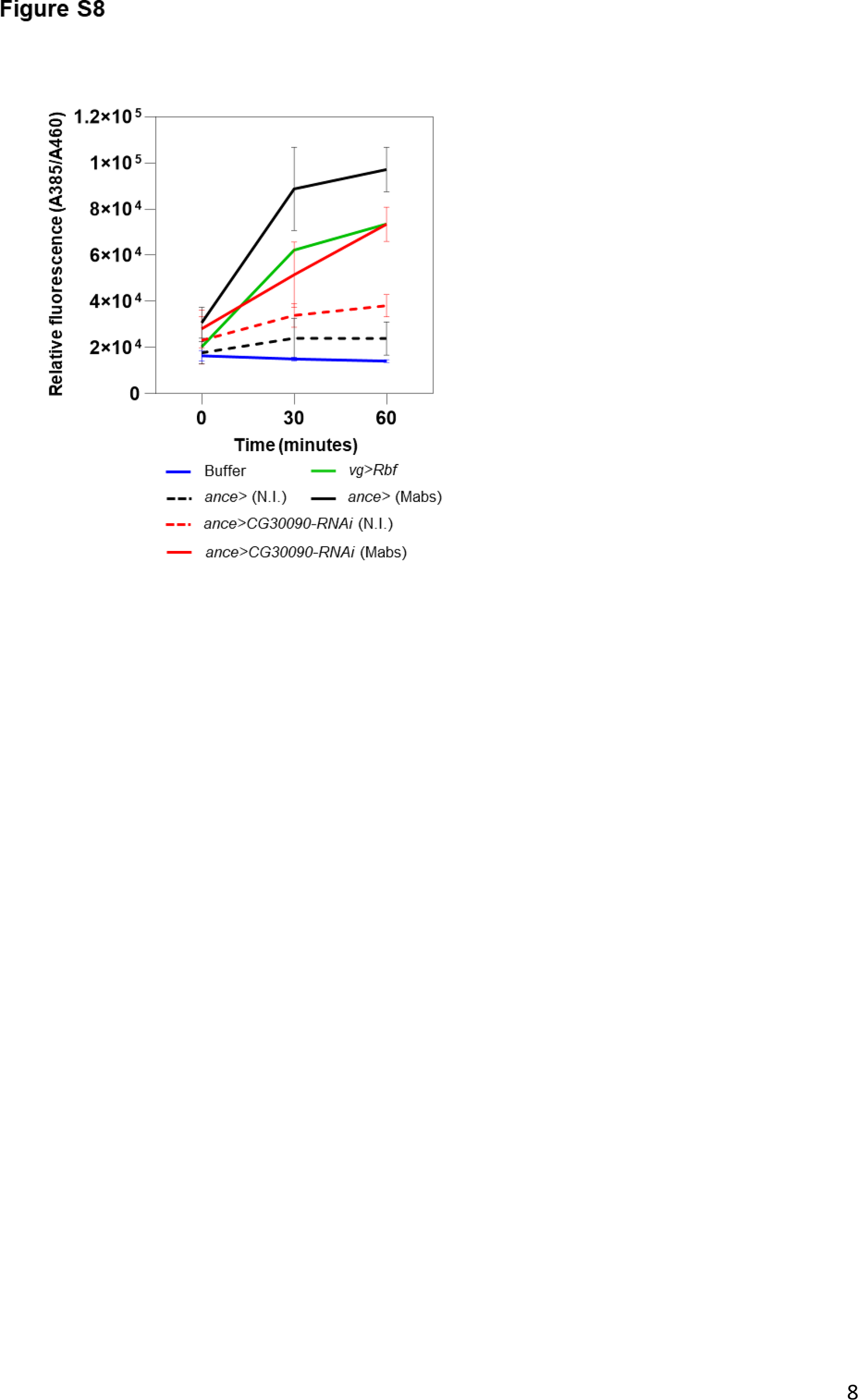

Supplement: S8 Fig — Kinetics of A385/A460 relative fluorescence released by the fluorochrome-conjugated caspase-3 substrate Ac-DEVD-AFC after incubation with buffer, vg>Rbf larval disc, hemocytes from non-infected (N.I.) or infected (Mabs) ance> and ance>CG30090-RNAi fly protein extracts on day 4 p.i.. Curves are representative of 3 independent experiments and the error bars represent the standard deviations. (TIF) [file ppat.1011257.s008.tif]
